# Organic acid production from potato starch waste fermentation by rumen microbial communities from Dutch and Thai dairy cows

**DOI:** 10.1186/s13068-018-1012-4

**Published:** 2018-01-25

**Authors:** Susakul Palakawong Na Ayudthaya, Antonius H. P. van de Weijer, Antonie H. van Gelder, Alfons J. M. Stams, Willem M. de Vos, Caroline M. Plugge

**Affiliations:** 1 Laboratory of Microbiology, Wageningen University & Research, Stippeneng 4, 6708 WE Wageningen, The Netherlands; 2 Thailand Institute of Scientific and Technological Research, 35 Mu 3, Khlong Ha, Amphoe Khlong Luang, 12120 Pathum Thani Thailand; 3 CEB-Centre of Biological Engineering, University of Minho, Campus de Gualtar, 4710-057 Braga, Portugal; 4 RPU Immunology, Department of Bacteriology and Immunology, University of Helsinki, Haartmaninkatu 3, 00014 Helsinki, Finland

**Keywords:** Lactate fermentation, Microbial communities, Renewable energy, Rumen fluid, Organic acids, Starch waste

## Abstract

**Background:**

Exploring different microbial sources for biotechnological production of organic acids is important. Dutch and Thai cow rumen samples were used as inocula to produce organic acid from starch waste in anaerobic reactors. Organic acid production profiles were determined and microbial communities were compared using 16S ribosomal ribonucleic acid gene amplicon pyrosequencing.

**Results:**

In both reactors, lactate was the main initial product and was associated with growth of *Streptococcus* spp. (86% average relative abundance). Subsequently, lactate served as a substrate for secondary fermentations. In the reactor inoculated with rumen fluid from the Dutch cow, the relative abundance of *Bacillus* and *Streptococcus* increased from the start, and lactate, acetate, formate and ethanol were produced. From day 1.33 to 2, lactate and acetate were degraded, resulting in butyrate production. Butyrate production coincided with a decrease in relative abundance of *Streptococcus* spp. and increased relative abundances of bacteria of other groups, including *Parabacteroides*, *Sporanaerobacter*, Helicobacteraceae, Peptostreptococcaceae and Porphyromonadaceae. In the reactor with the Thai cow inoculum, *Streptococcus* spp. also increased from the start. When lactate was consumed, acetate, propionate and butyrate were produced (day 3–4). After day 3, bacteria belonging to five dominant groups, *Bacteroides, Pseudoramibacter*_*Eubacterium*, *Dysgonomonas*, Enterobacteriaceae and Porphyromonadaceae, were detected and these showed significant positive correlations with acetate, propionate and butyrate levels.

**Conclusions:**

The complexity of rumen microorganisms with high adaptation capacity makes rumen fluid a suitable source to convert organic waste into valuable products without the addition of hydrolytic enzymes. Starch waste is a source for organic acid production, especially lactate.

**Electronic supplementary material:**

The online version of this article (10.1186/s13068-018-1012-4) contains supplementary material, which is available to authorized users.

## Background

Consuming fossil fuels has become a significant concern not only because resources are depleting but also because of the resulting pollution and carbon dioxide formation that contribute to global warming. However, there is also a worldwide increasing energy demand. Renewable instead of fossil sources for the production of energy and biochemical building blocks are thus of interest. Turning waste into energy carriers and valuable products is currently one of the promising sustainable options, especially since waste disposal requires energy (incineration) or space (landfills). Organic waste includes food and fibre processing by-products, fruit, vegetable waste, garbage, sewage sludge, cattle manure and/or industrial waste [1]. All these materials have no or low value and do not impact the food-value chain [2]. The possibility to conserve energy from waste and/or biomass is a strong motivation to further develop biobased processes and is in line with recently developed strategies that aim to use anaerobic mixed cultures for the conversion of organic feeds into carboxylates, including volatile fatty acids (VFAs) and/or organic acids (OAs) [3, 4]. Such a carboxylate platform may gain higher value of production efficiency than, for example, biogas (methane) formation [3], since VFAs and/or OAs can be used as biobased building-block chemicals [4, 5]. Demand for lactate, for instance, continuously increases due to its various applications as an acidulant, flavour enhancer or food preservative agent in addition to the production of base chemicals and for polymerisation to biodegradable polylactic acid (PLA) [6, 7]. PLA is a biodegradable plastic derived from lactate, and PLA is already available at the industrial scale. Microbial lactate fermentation has advantages over the chemical lactate synthesis in terms of obtaining purity where the chemical synthesis always results in a racemic mixture of lactate [2, 8]. It is important to select raw materials with suitable criteria such as high lactate production yield, rapid fermentation, low cost, low by-product formation and all-year-round availability for industrial lactate production [2]. As the world’s second-most abundant biopolymer, starch serves as food, feed and other industrial applications [9], leading to a large amount of starch waste and starch residues. Starch residues from various sources, such as barley, cassava, corn and/or potato, can be used for VFA and/or OA production [8, 10] and can meet industrial needs. Starch is the main component of the potato tuber with 80% of the dry solids and 20% of the total mass which can be used as a carbon source for microorganisms. Many studies have been conducted using potato starch and/or potato starch waste as a substrate in anaerobic digestion [10–13]. Mostly, those studies use starch waste to produce biogas, OAs or animal feed components. However, little attention has been paid to the microbial community involved in the OA production from starch waste.

The efficiency of the anaerobic digestion process (in terms of production and digestion yields) relies on many factors such as chemical parameters (pH, nutrient content), physical parameters (temperature, mixing) and biological factors (biomass) [14]. The choice of inoculum is an important factor. Single or mixed cultures have been used in organic acid production; however, using pure culture mostly requires pre-treatment processing, including sterile operation, which increases the production costs.

Rumen fluid has been used as an inoculum in biotechnological processes to improve municipal solid waste treatment in anaerobic digestion [15] and to increase hydrolysis of cellulosic organic material [16, 17]. Using a rumen-derived inoculum, which harbours high microbial hydrolytic activities, could reduce the pre-treatment costs in anaerobic digestion since there is little or no requirement to add hydrolytic enzymes [18]. The rumen contains a large number of microorganisms with enormous diversity, of which includes at least 50 bacterial genera (10^10^–10^11^ cells ml^−1^), 25 genera of ciliate protozoa (10^4^–10^6^ cells ml^−1^), 6 genera of fungi (10^3^–10^5^ zoospores ml^−1^), 11 genera of methanogenic archaea (10^9^ cells ml^−1^) and bacteriophages (10^8^–10^9^ phages ml^−1^) [19, 20]. Rumen microorganisms are naturally involved in the degradation of carbohydrates and lignocellulosic biomass to a variety of VFAs and OAs [21]. In this view, using rumen fluid as an inoculum is an attractive option for OA production.

The microbial community composition in the cow rumen depends on the feed composition [18]. In tropical countries, such as Thailand, the feed mainly consists of crop residues, which can be lignocellulosic agricultural by-products of rice, corn, cassava, cereal straws, sugarcane, groundnut and/or pineapple processing industries [22]. On the other hand, cows in temperate countries, such as the Netherlands, are regularly fed with wheat and corn silage. Therefore, it is interesting to investigate and compare the OA production profiles and the microbial communities from both rumen inoculum sources.

We studied organic acid production from starch waste using rumen fluid as the inoculum and investigated the microbial composition shift during the process. Two different sources of rumen fluid obtained from fistulated cows in the Netherlands and Thailand were used.

## Methods

### Reactor setup

Fermentations were performed in batch mode using 1-l dished-bottom reactors (Applikon, Delft, The Netherlands) with a working volume of 0.9 l and controlled by an ADI 1010 Bio-controller and an ADI 1025 Bio-console (Applikon, Delft, the Netherlands). Temperature of both cow rumen-inoculated reactors was controlled at 39 °C to mimic conditions in the cow rumen. The pH was maintained at 7.0 ± 0.4 by automatic titration with a sterile solution of 3 M Na_2_CO_3_. The stirrer speed was set at 120 rpm to keep the starch waste homogeneous. The reactors were continuously sparged with 80:20 N_2_/CO_2_ at a flow rate of 2.6 l h^−1^ to ensure anaerobic conditions. Typically, the reactors were operated for 8 days and daily samples were taken during the fermentation.

### Inoculum

Bovine rumen fluid (500 ml) was collected from two fistulated Holstein cows from two different locations: The Netherlands and Thailand. The cows were aged between 4 and 5 years at the sampling period. The Dutch cows were fed with a high-grain diet with mainly corn (maize) and grass at Wageningen University’s research farm in the Netherlands (Additional file 1: Table S1). The Thai cows were fed with mainly pineapple peel at the Charoen Pokphand Test Farm, Chon Buri in Thailand (Additional file 1: Table S1). After sampling, the rumen fluid was quickly filtered through two layers of cotton cloth in ambient air. The filtered rumen fluid was then transferred into a sterilised CO_2_-flushed anaerobic bottle and was kept at 4–10 °C until use. The Thai rumen fluid was shipped to the Netherlands in a cooled container at approximately 4 °C. Samples of both rumen fluids were used to inoculate the reactors (1% v v^−1^).

### Medium composition

A bicarbonate-buffered anaerobic mineral medium (BM) was prepared as described by Plugge [23] supplemented with (l^−1^): 0.1 g yeast extract, 0.005 g hemin, 0.05 g vitamin K1 and 0.5 g l-cysteine-hydrochloride. Starch-containing waste was obtained from an Avebé potato factory (Foxhol, The Netherlands) and was used as substrate. The starch waste was air dried at 80 °C for 32 h, crushed to small pieces and sieved with 1-mm pore-size sieve. The dried starch waste contained 61% (w w^−1^) starch according to the analysis of Nutricontrol (Veghel, The Netherlands) (Additional file 2: Table S2). Dried starch waste (7%, w v^−1^) was added to the reactors as carbon and energy sources. After autoclaving, the sterile reactors with medium were continuously flushed with sterile 80:20 N_2_/CO_2_.

### Sampling

As starch waste has a high viscosity, the fermentation broth was pumped through a loop with a butyl-rubber stopper to facilitate anaerobic sampling. Ten-millilitre liquid samples were aseptically collected and transferred directly into sterile-anaerobic serum bottles. Each sample was divided into three portions. One portion of 6 ml was transferred to a 10-ml sterile-anaerobic serum bottle and stored at − 20 °C for molecular analysis. A second portion of 2 ml was transferred to an Eppendorf tube for organic acid measurement. A third portion of 2 ml was transferred to a sterile-anaerobic serum bottle for CFU (colony-forming units) counts.

### Deoxyribonucleic acid (DNA) extraction

Genomic DNA was extracted from the pelleted biomass using a Fast DNA Spin kit for soil (MP Biomedicals Santa Ana, CA) according to the manufacturer’s instructions. DNA quantity of all samples was determined by a Nanodrop 1000 (Nanodrop Technologies, Wilmington, DE) and integrity was examined by gel electrophoresis on the 1% (w v^−1^) agarose gel.

### Denaturing gradient gel electrophoresis (DGGE) analysis

DGGE analysis was used to visualise the population dynamics in both reactors over time. Bacterial 16S rRNA V6–V8 regions were amplified with the universal bacterial primers GC-968F and 1401R [24] using the Phire Hot start II Polymerase (Thermo Fisher Scientific, Waltham, MA). Bacterial amplicons were generated with a G-Storm cycler (G-storm, Essex, UK) using a pre-denaturing step at 95 °C for 5 min, followed by 35 cycles at 95 °C for 20 s, 56 °C for 40 s, 72 °C for 40 s and a post-elongation step of 10 min at 72 °C. The forward primer had a GC clamp of 40 bp attached to the 5′-end as used by Yu et al. [25]. DGGE analysis was performed as described by Martín et al. [26] in a DCode TM system (Bio-Rad Laboratories, Hercules, CA) at 60 °C for 16 h with a denaturing gradient of 30:60 percent gradients according to [25]. After electrophoresis, gels were silver-stained as described by Sanguinetti et al. [27] and scanned.

### Pyrosequencing analysis

Based on the bacterial DGGE profiles from both reactors, samples were selected to determine the relative abundance of the bacteria using 454-pyrosequencing analysis.

The genomic DNA obtained from the previous step was diluted to obtain DNA concentrations between 10 and 20 ng µl^−1^ as templates. The V1–V2 regions of bacterial 16S rRNA genes were amplified using forward primer 27F-DegS [28] and an equimolar mix of two reverse primers: 338R-I and 338R-II [29]. The forward primer was extended with titanium adapter A and an eight-base specific barcode [30] at the 5′-end and the reverse primers were appended with titanium adapter B at the 5′-ends. The polymerase chain reaction (PCR) amplification and the purification of the amplicons were performed as previously described by Timmers et al. [31]. The DNA concentration of all PCR products was measured using Qubit 2.0 Fluorometer (Thermo Fisher Scientific, Waltham, MA) and was then mixed together in equimolar amounts. Pooled samples were loaded on an agarose gel and bands were excised, purified and quantified using the protocol of Timmers et al. [31]. The purified pooled samples were sent to GATC Biotech Company (Konstanz, Germany) for pyrosequencing on the 454 Life Science GS-FLX platform.

Pyrosequencing data were analysed using a workflow based on Quantitative Insights into Microbial Ecology (QIIME) 1.7.0 pipeline [32]. The reads were filtered and the Operational Taxonomic Units (OTUs) were identified with a cut-off value of 97% identity by USEARCH algorithm version 6.1 [33]. Representative sequences from OTUs were aligned using PyNAST [34] against with SILVA reference database version 118e [35] for taxonomic classification. Chimeric OTUs were identified and removed using QIIME’s ChimeraSlayer method [36].

### Bacterial CFU counts during fermentation process and isolation of fermentative bacteria

Total viable bacterial counts in both reactors were determined by colony-forming unit (CFU) plate counts over the entire incubation period. The samples were homogenised by vortexing. Next, tenfold dilutions in liquid Reinforced Clostridial Medium (RCM) in dilution 10^−1^–10^−10^ and 20 µl of each dilution was spread (in triplicate) on RCM agar plates (1.2% agar). The plates were incubated in a jar containing AnaeroGen™ sachets (Oxoid-Thermo Scientific; Hampshire, UK) to create and maintain anaerobic conditions and incubated at 39 °C for 3–5 days. After incubation, the colonies were counted and log_10_ CFUs ml^−1^ were calculated. Colonies with different morphology were selected and further purified using the streak plate technique on RCM agar plates until pure cultures were obtained. The pure cultures were then grown in the RCM liquid medium. Cell morphology of the cultures was observed using a light microscope (Leica DM 2000; Buffalo Grove, IL) to confirm the purity.

### Identification of the isolated bacteria

Genomic DNA of each pure strain was isolated using the Fast Spin kit for soil (MP Biomedicals; Santa Ana, CA) following the manufacturer’s instructions. The 16S rRNA gene of each isolate was amplified by PCR using bacterial-universal primers 27f and 1492r [37]. PCR mixture contained: 2 µl of DNA template, 0.25 µl Gotaq DNA Polymerase Kit (Promega; Medison, WI), 1 µl dNTPs, 1 µl of each primer, 10 µl PCR buffer and 34.75 µl PCR water. The PCR programme was started with a denaturing step at 95 °C for 5 min, and continued with 35 cycles consisting of 95 °C for 30 s, 52 °C for 40 s and 72 °C for 90 s, and the last step of extension at 72 °C for 7 min. PCR products were purified and sequenced at GATC (Konstanz, Germany). The 16S rRNA gene sequences of the isolates were checked for reading errors, trimmed and aligned using the programme DNA Baser Sequence Assembler v4 (Heracle BioSoft S.R.L, Arges, Romania), and then the partial sequences of 16S rRNA genes were blasted against the NCBI online database.

### Statistical analysis

Principal Coordinate Analysis (PCoA) using weighted unifraction and Unweighted Pair Group Method with Arithmetic mean (UPGMA) were performed using the QIIME 1.7.0 pipeline to show the relationship of bacterial communities at different time points in the starch waste fermentation from both reactors. Multivariate analyses were performed with the CANOCO V 5.0 software [38] using the pyrosequencing results and OA production profiles from both Dutch and Thai reactors. OA production profiles were used as ‘environmental’ variables and the bacterial diversity (at genus-like level) as ‘species’ variables. First, principal component analysis (PCA) was used to visualise the overall correlation between all variables at different time points in both Dutch and Thai reactors, separately. Then, selected OAs based on primary OAs in each reactor were analysed to reveal the relationship between variables using redundancy analysis (RDA). The significance test for RDA was performed by Monte Carlo permutation (499 times). The significant correlations between bacterial groups and operational conditions in each reactor were calculated with Ranked Spearman correlation by IBM SPSS Statistics version 23.

### Analytical methods

During fermentations, off-gas composition was automatically monitored every hour using a Compact GC (Interscience, Breda, The Netherlands), equipped with a Carboxen 1010 PLOT column and a Micro thermal conductivity detector, using helium with pressure flow 80.0 kPa as a carrier gas to quantify H_2_ and CH_4_ production. The production of OAs was quantified at 24-h intervals for over 16 days by HPLC (Thermo Scientific, Breda, The Netherlands), as described in [39].

### Nucleotide sequence accession numbers

The 16S rRNA gene sequences obtained from the isolates were deposited in the NCBI database and are available under Accession Numbers MF581503–MF581530. The 16S rRNA NGS sequences were deposited at the EMBL database and are available under Accession Numbers ERS1983120–ERS1983133.

## Results and discussion

### Starch waste fermentation in Dutch and Thai reactors

**Fig. 1:**
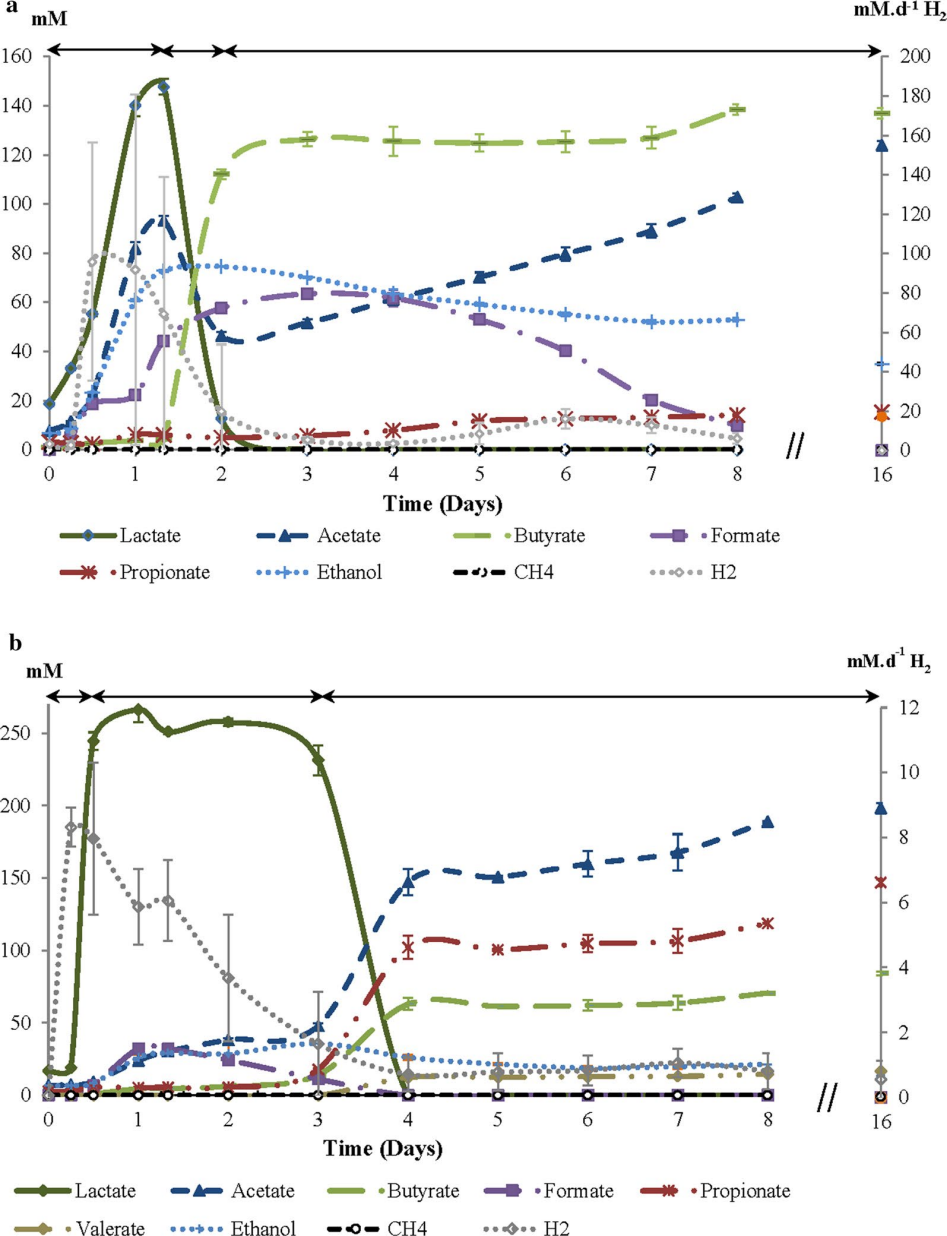
Production profiles in starch waste fermentation using the Dutch (**a**) and Thai (**b**) rumen fluids as inoculum in anaerobic reactors. H_2_ is shown on the secondary axis. The arrows indicate three stages in the fermentation

The reactors were fed with starch waste and the fermentation process was followed for 16 days, with a focus on the first 8 days. In both reactors, lactate formation immediately started after inoculation with rumen fluid, and gradually changed to mixed acid fermentation after a few days (Fig. 1).

Lactic acid production is common and has been shown in many starch or starchy fermentation studies. For instance, lactate was the major product in potato peel waste fermentation [40], and lactate and acetate the main products in maize silage fermentation [41]. Lactic acid-producing bacteria are the first and the most rapidly growing microbes in the starch fermentation.

In the Dutch reactor, three stages could be identified in the fermentation (Fig. 1a; Additional file 3: Table S3). During the first stage (day 0–1.33), lactate, acetate and ethanol rapidly accumulated to 148, 93 and 73 mM, respectively. In the second stage (day 1.33–2), lactate and a part of acetate were consumed and converted to butyrate (112 mM). In the third stage (after day 2), butyrate and ethanol remained constant, whilst acetate increased further until 103 mM (at day 8). Formate also increased from 22 (day 1) to 64 mM (day 3) and then decreased again to about 10 mM (day 8). Propionate increased from day 1 to 8 from 6 to 14 mM.

Also in the Thai reactor, three stages in the fermentation pattern could be identified (Fig. 1b; Additional file 4: Table S4). During the first stage (day 0–0.5), lactate rapidly increased from 17 to 245 mM. In the second stage (day 0.5–3), lactate remained stable at 250 ± 13 mM, whereas acetate, butyrate, propionate and ethanol increased further to 48, 14, 18 and 36 mM, respectively. Formate increased from 7 (day 0.5) to 32 mM (day 1) and was completely consumed at day 3. In the early third stage (day 3–4), lactate (231 mM) was completely and ethanol (10 mM) was partially consumed. Increasing the levels of acetate, propionate and buyrate detected on day 4–147, 102 and 63 mM, respectively. From day 4 to day 16, the product pattern remained constant (Additional file 4: Table S4).

The maximum yields of lactate were 0.3 (Dutch) and 0.6 (Thai) g g^−1^ starch and the highest lactate concentrations were 13 and 25 g l^−1^ (calculated using 90 g M^−1^ as the MW of lactate) obtained from 42 g l^−1^ of starch present in the starch waste. This gave 31 and 60% lactate yield at day 1.33 and 2 in the Dutch and Thai reactors, respectively. In the calculations, it was considered that starch waste contains 61% starch (Additional file 2: Table S2). The lactate production that was observed from the Thai reactor was somewhat higher than that reported in a previous study which delivered 50% lactate yield (or the yield of lactate was 0.4 g g^−1^ starch present in cassava fibrous residue), where about 30 g of lactate was produced from 60 g of starch present in 100 g of cassava fibrous residue using a pure culture of *Lactobacillus plantarum* MTCC 1407 (2% v v^−1^ of the inoculum) [42, 43]. In another study in which an undefined mixed culture (2% v v^−1^ of inoculum) was used in potato peel waste fermentation with addition of hydrolytic enzymes, lactate production of 14.7 g l^−1^ and a yield of 0.7 g g^−1^ starch (calculated with 34.3% starch in the initial substrate loading) were observed [40], which is higher than our yield. Our study was conducted based on using the ruminal mixed culture (1% v v^−1^ of inoculum) without adding any hydrolytic enzymes or performing a pre-treatment step. Notably, the production yield using different wastes cannot be accurately compared, and additionally, the fermentation process has been influenced by other factors: fermentation conditions, type and amount of inoculum, and the composition of the waste materials, for instance. However, this robust lactate production from starch waste challenges us to further optimise lactate production in the future. Natural producers of lactate are very efficient, but pure cultures and synthetic communities have drawbacks such as high nutrient requirement. Efforts to further improve LA yield and engineer lactate production by redirecting the carbon flow for LA production have been reported. Pyruvate is the end product of glycolysis and can be further metabolised either by a pyruvate dehydrogenase complex (Pdh, EC 1.2.4.1) to acetyl-coenzyme A or by pyruvate decarboxylase (Pdc, EC4.1.1.1) to acetaldehyde and subsequently to ethanol. In previous works, it has been shown that the expression of a heterologous lactate dehydrogenase (Ldh, EC 1.1.1.27) gene introduces a new and alternative pathway for NAD^+^ regeneration, allowing a direct reduction of the intracellular pyruvate to lactate [44]. CRISPR–Cas-based tools have been presented as the potential next-generation toolkit for prokaryotic metabolic engineering, for genome editing and expression control, and have enabled fast, easy and accurate strain development for established production platform organisms, such as *Escherichia coli* and *Saccharomyces cerevisiae* [45]. Future dedicated research could focus on development of the CRISPR–Cas-based tools for improved LA production.

In both Dutch and Thai reactors, methane was not detected in the beginning of the fermentation process, but only appeared in trace amounts after 7 days in the headspace (0.23 and 0.018 mM d^−1^, respectively), whilst increasing after 2 weeks (4.4 and 11.1 mM per day, respectively at day 16: data not shown). For hydrogen, 95.6 and 9.2 mM per day out flow of the Dutch and Thai reactors, respectively, were detected at the start (Day 0.5) of the fermentation process (Fig. 1). Notably, there was an unknown peak in our HPLC chromatogram (retention time 8.15), which was detected from both reactors and we could not identify the compound.

### Bacterial CFUs and isolation of bacteria

In both starch waste fermenting reactors, total bacterial counts increased up to 10.3 (± 0.1) log_10_ CFU ml^−1^ (standard deviation: SD) in 24 h (Additional file 5: Table S5). From day 1 until 8, the average total bacterial counts were 7.6 (± 1.1) and 8.6 (± 1.1) [log_10_ CFU ml^−1^ (SD)] and between day 8 and 16, the total bacterial counts decreased to 4.8 (± 0) and 6.4 (± 0) log_10_ CFU ml^−1^ for the Dutch and Thai reactors, respectively.

Ten different bacterial strains (28 in total) were isolated from both reactors based on different colony morphologies on RCM agar medium during the fermentation process (Additional file 6: Table S6). Mainly, *Streptococcus* spp. were isolated from the first fermentation stage, and other fermentative bacteria (*Enterococcus faecium*, *Enterococcus gallinarum*, *Escherichia fergusonii*, *Lactobacillus plantarum*, *Enterococcus durans*, *Clostridium sporogenes* and *Eubacterium limosum*) were isolated from the second and third fermentation stages. *Lactobacillus* spp. were isolated only from the Thai reactor. The majority of these isolates are lactic acid bacteria (LAB). All of them were Gram-positive bacteria except *Escherichia fergusonii,* which was isolated at the third fermentation stage from the Dutch reactor. Lactate was the principal product of the first fermentation stage, and is also known for its antibacterial properties. Lactate penetrates the cytoplasmic membrane, hence lowering the intracellular pH as well as disintegrating the outer-membrane in Gram-negative bacteria [46]. Gram-positive bacteria have a thicker cell wall than Gram-negative bacteria, which enables them to sustain harsh conditions, such as high concentrations of lactate in the medium, which explains why mostly Gram-positive bacteria were isolated from this fermentation.

### Bacteria (DGGE) profiles

**Fig. 2:**
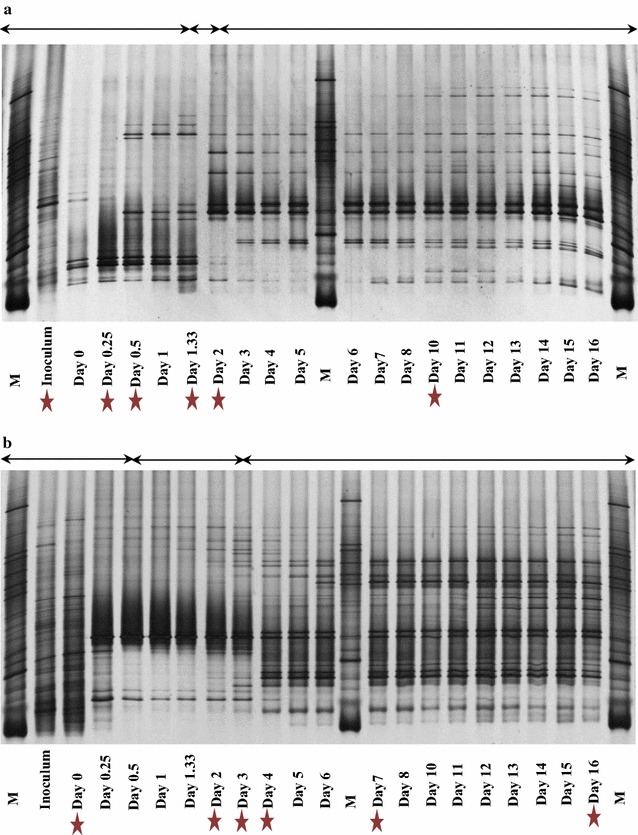
16 rRNA based DGGE profiles of bacteria involved in starch waste fermentation in reactors with (**a**) Dutch and (**b**) Thai cow rumen fluids. ‘M’ refers to marker. Asterisks indicate samples that were used for NGS analysis. The arrows indicate three stages in the fermentation

Bacterial DGGE profiles from the Dutch reactor could be grouped into three patterns (Fig. 2a). These patterns matched the three stages of the fermentation profiles (Fig. 1a). In the first stage (day 0–1.33), bands were visible with increasing intensity. The band pattern then shifted during stage 2 (day 1.33–2). Finally, in the third stage, the number of bands increased further, which indicated an increased bacterial diversity. Then, the microbial diversity remained stable until the end of the run (day 16).

Also in the Thai reactor, the bacterial DGGE profile could be grouped into three patterns that matched the three stages of the fermentation (Fig. 2b). In the first stage (day 0–0.5), one very dense band was visible amidst a variety of bands. In the second stage (day 0.5–3), banding patterns were less diverse and one band appeared with high intensity (Fig. 2b). Finally, in the third stage, the number of bands increased, and then remained stable until the end of the run (day 16).

Notably, the intense bands from both reactors lined in the same position, which might possibly represent *Streptococcus* spp., since only this member shared the same OTU and was highly abundant during the fermentation process (Additional file 7: Figure S1).

### Bacterial community analysis

16S rRNA gene amplicon pyrosequencing of the V1–V2 regions of the 16S rRNA gene of selected samples (based on the different pattern on DGGE profiles) from both reactors was used to analyse the bacterial communities during starch waste fermentation. After quality control, 228,106 sequence reads could be translated into 253 OTUs (Additional file 8: Table S7). OTUs were then identified with a cut-off value of 97% identity and were assigned to 30 phyla, 53 classes, 101 orders, 155 families and 253 genera. Taxa with relative abundance < 1% and unclassified groups were termed as ‘others’.

### Comparing the bacterial composition between two inocula

At phylum level, Bacteroidetes was the most abundant (66% relative abundance) in the Dutch rumen inoculum, followed with Firmicutes and Cyanobacteria (22 and 6% relative abundances, respectively), whereas Firmicutes was the most abundant (41% relative abundance) followed with Bacteroidetes and Proteobacteria (34 and 10% relative abundances, respectively) in the Thai rumen inoculum (Additional file 9: Figure S2).

**Fig. 3:**
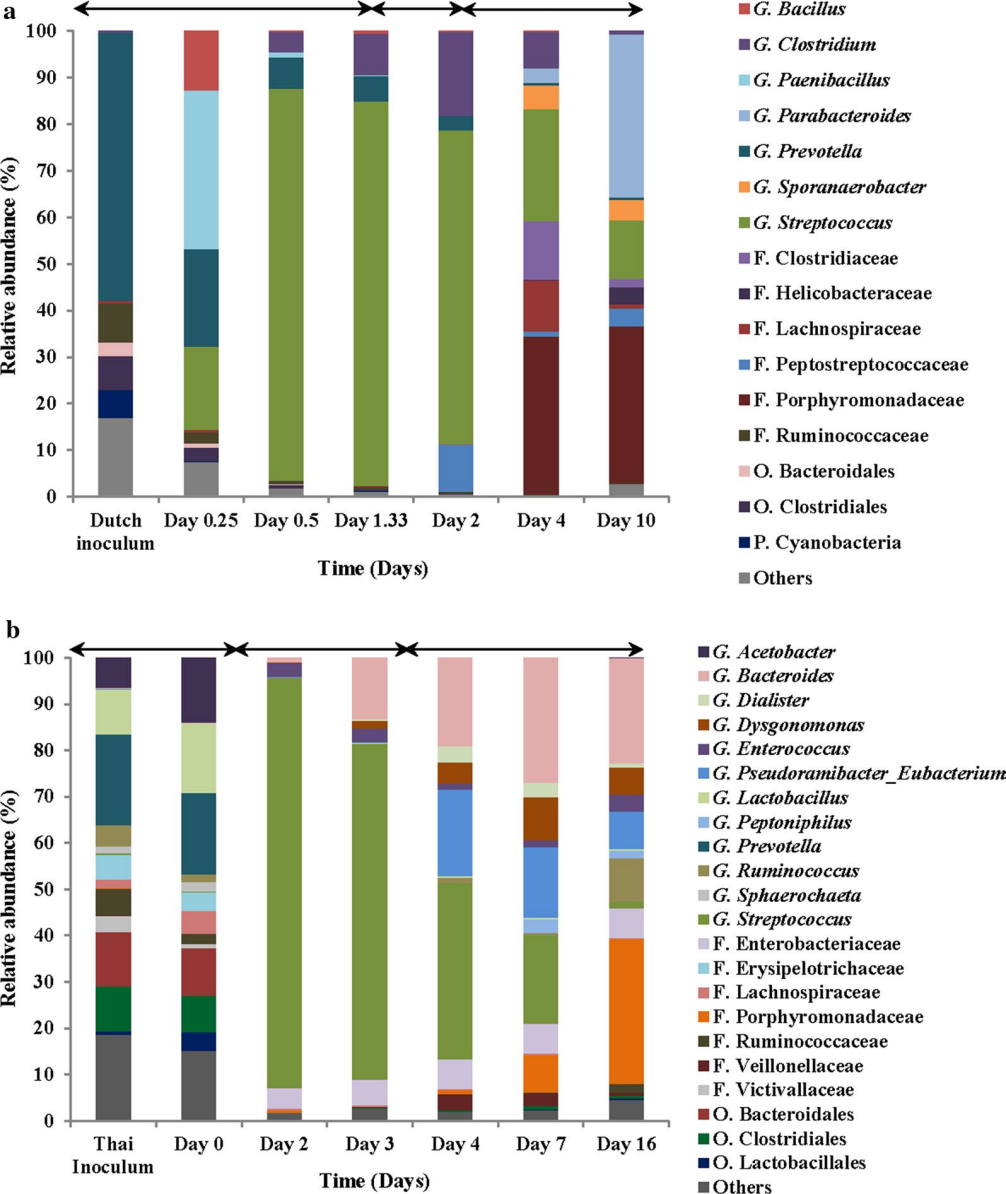
Relative abundance of bacterial communities (genus-like level) in the starch waste fermentation using Dutch (**a**) and Thai (**b**) cow rumen. Taxa with relative abundance < 1% in all samples were grouped into the category ‘others’. *P* phylum, *O* order, *F* family, *G* genus. The arrows indicate three stages in the fermentation

At genus level, the microbial diversity in the Thai rumen inoculum was higher (3 orders, 7 families and 12 genera) compared to the Dutch rumen inoculum (1 phylum, 2 orders, 6 families and 7 genera) (Fig. 3). In Dutch rumen inoculum, *Prevotella* was the most abundant (58% relative abundance) followed by Ruminococcaceae and Clostridiales (8 and 7% relative abundance, respectively) (Fig. 3). In the Thai rumen inoculum, *Prevotella* was also the most abundant (20% relative abundance) followed by members of *Bacteroidales*, *Clostridiales* and *Lactobacillus* (12, 10 and 10% relative abundance, respectively) (Fig. 3b). Notably, the relative abundance of *Prevotella*, which is commonly known as the dominant amylolytic species in rumen of the high-grain diet-fed cows [47], was rather different between Dutch and Thai inocula (58 and 20% relative abundances, respectively). This can be explained by the difference in feed composition as the Dutch cows were fed 6.5 kg dry matter intake per day of maize silage, and no maize diet was fed to the Thai cows (Additional file 1: Table S1). On the other hand, *Lactobacillus* and *Acetobacter* (10 and 6% relative abundances, respectively) were only detected in the Thai rumen inoculum, of which the cows were fed with 3.3 kg dry matter intake per day of pineapple peel (Fig. 3; Additional file 7: Figure S1). Indeed, *Lactobacillus* was only isolated from the Thai reactor. Various LAB, especially *Lactobacillus* were isolated from pineapple [48] and one of the common diseases in pineapple is ‘marbling disease’ which is caused by acetic acid bacteria such as *Acetobacter* spp. [49]. These results show that the microbial community composition in the rumen is strongly dependent on the feed composition [18] and may affect the fermentation profiles in a reactor.

### Comparing the bacterial composition between the two reactors during the fermentation process

The bacterial community composition at the phylum level from both reactors is shown in Additional file 9: Figure S2. Bacteroidetes and Firmicutes were most represented in both communities. The work of Gou revealed that in a starch-fed reactor, two bacterial phyla, Spirochaetes and Firmicutes (*Streptococcus*), were mainly responsible for starch degradation [50]. Interestingly, in the Thai rumen community, Proteobacteria remained in the community throughout the reactor run. In the Dutch reactor, OTUs affiliated with Proteobacteria were detected in stage 3 (day 10), albeit at low levels.

#### The Dutch reactor

In the Dutch reactor, *Prevotella* spp. (58% relative abundance) were dominant, but gradually decreased to less than 3% relative abundance at day 4 (Fig. 3a). At day 0.25, genus: *Streptococcus* (18%)*, Paenibacillus* (34%) and *Bacillus* (13%) increased in relative abundances, but after day 0.25 to 2, *Streptococcus* spp. became dominant (~ 80% relative abundance). From day 1.33 until day 2 (second stage), *Streptococcus* spp. decreased to 67%, whereas the relative abundance of *Clostridium* and Peptostreptococcaceae (18 and 10%, respectively) increased. In the third stage (day 4), the relative abundance of Clostridiales member remained stable with Clostridiaceae (13%) and *Clostridium* (8%). On day 4, Porphyromonadaceae appeared and remained until day 10 with 34% relative abundance. From this family at genus level, *Parabacteroides* was also detected and its relative abundance increased 3% at day 4 to 35% at day 10. The remaining fraction of Porphyromonadaceae (35% relative abundance) could not be identified to a deeper phylogenetic level.

#### The Thai reactor

As observed in the Dutch reactor, *Prevotella* (18% relative abundance) members were also abundant in the Thai reactor at day 0 and decreased to less than 1% relative abundance at day 2 (Fig. 3b). At day 2, *Streptococcus* spp. (~ 90% relative abundance) were highly abundant and remained dominant until day 4 (38% relative abundance). At day 2, the second-most abundant group was members of Enterobacteriaceae (8%), in which *Enterococcus* had 3% relative abundance. From day 3 to 4, the relative abundance of *Pseudoramibacter*_*Eubacterium* spp. increased (19%), whereas *Streptococcus* spp. gradually decreased (35%) until day 16 (2%). After day 4, *Bacteroides* (27%), *Pseudoramibacter_Eubacterium* (15%) and Porphyromonadaceae (17.4%) became dominant. In the family Porphyromonadaceae, only one genus, *Dysgonomonas*, was identified and its relative abundance (9%) was highest at day 7. Porphyromonadaceae members gradually increased and became the most abundant group (31%) in the reactor at day 16.

### The relationship between starch waste fermentation and bacterial communities

OA production profiles mainly depend on the type of substrates and source of the microbial inoculum [11, 51]. Using activated sludge from three different municipal wastewaters and potato peel wastes as substrates, lactate production was observed and bacteria of the genus *Lactobacillus* prevailed (> 96%) in all three incubations, even though they were not abundant (0.1%) in the seed sludges [51].

In our study, *Streptococcus* was detected only in small amounts in Dutch and Thai rumen inocula (0.03 and 0.3% relative abundances, respectively), but became highly abundant (84 and 89% relative abundances, respectively) during starch waste fermentation. *Streptococcus* was important for fast and efficient lactate (up to 250 mM) production during the first stage (0–1.33 days) in both reactors. Lactate was then the substrate for secondary fermentation to produce acetate, butyrate and propionate. OA production profile and bacterial composition of both reactors were different in the second part of the fermentation.

Principal component analysis (PCA) using a weighted Unifrac plot and grouping tree analysis revealed that all time points separated the Dutch and Thai bacterial communities (Additional file 10: Figure S3a). During the first stage of the fermentation process (day 0–2), the Dutch reactor communities clustered closely together. In stage 3 (day 4 and 10), they clearly developed into different communities. Communities from the Thai reactor directly separated from the inoculum and day 0, indicating growth of a dedicated bacterial community after the starting point of the fermentation process (Additional file 10: Figure S3b).

The abundance of the top 33 bacterial OTUs from the two reactors was also plotted in a heat map (Additional file 7: Figure S1). Overall, the OTUs from both reactors were different and only the genera *Prevotella* and *Streptococcus* were shared. *Prevotella* members were abundant at the start of the fermentation and decreased in time. Members from three families: Porphyromonadaceae, Peptostreptococcaceae and Enterobacteriaceae were shared and became abundant in the last stage of both reactors.

**Fig. 4:**
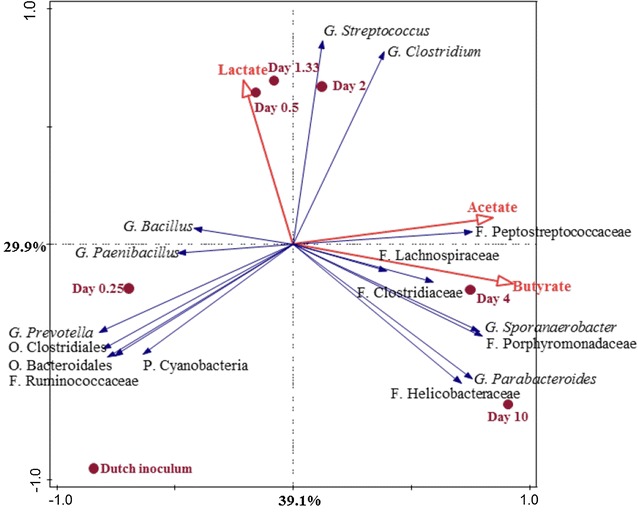
Redundance analysis triplot showing the relationship between the top 16 genus-like level phylogenetic groupings of the OTUs and the environmental variables explaining the variance with time in the Dutch reactor. Sampling days are shown as filled circles (●). Environmental variables or selected fermentation products are represented by red arrows. Bacterial community at genus-like groups with the level, i.e. phylum (P), order (O), family (F) or genus (G) are represented as blue arrows. The arrows indicate the direction in which the relative abundance increases. Length of arrows is a measure of fit. The environmental variable arrows (in red) approximated the correlation between species and an environmental variable. The further a product falls in the direction indicated by an arrow, the higher the correlation. Both axes together explained 69% of the total variance in the dataset

**Fig. 5:**
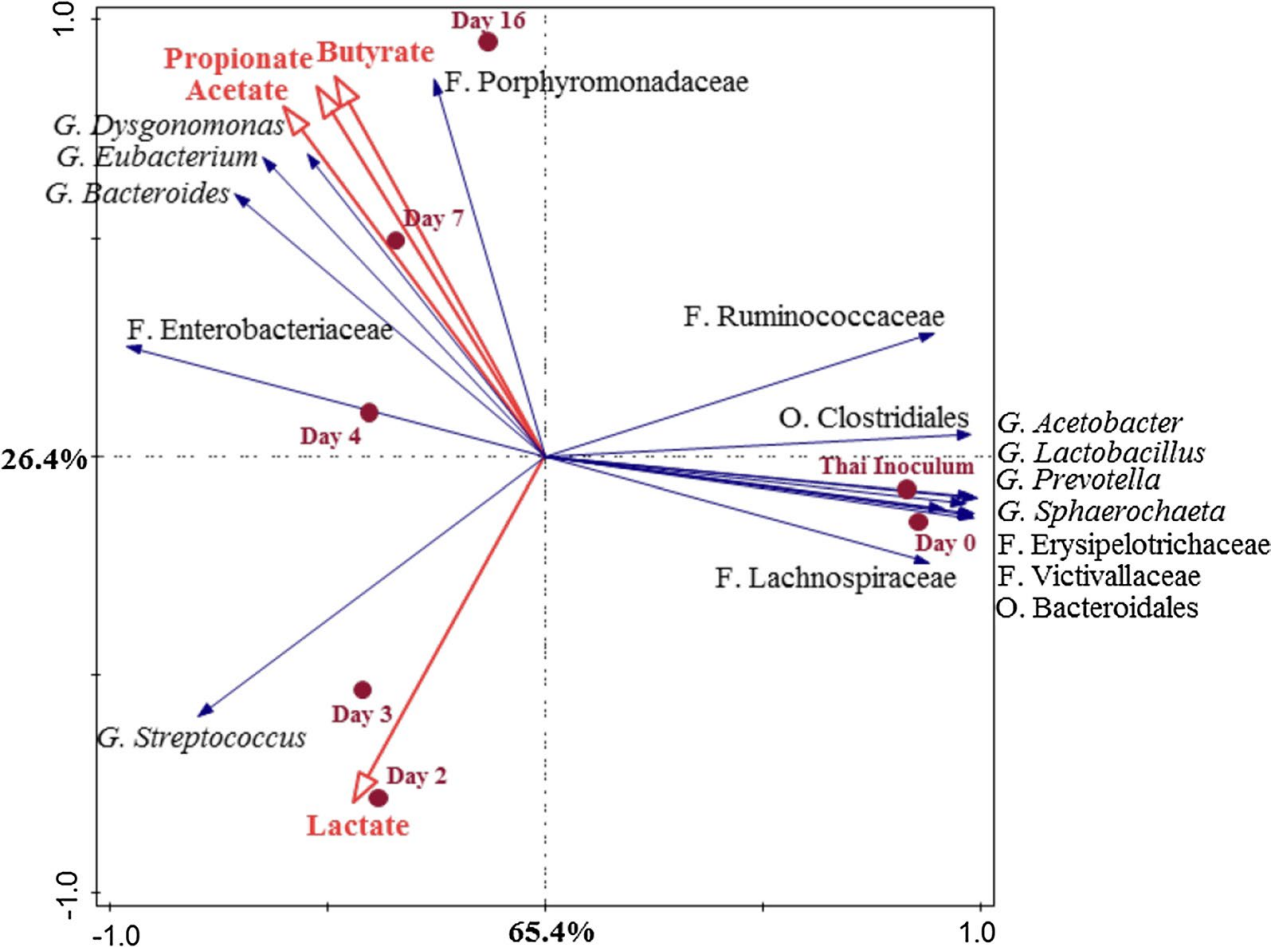
Redundance analysis triplot showing the relationship between the top 16 genus-like level phylogenetic groupings of the OTUs and the environmental variables explaining the variance with time in the Thai reactor. Sampling days are shown as filled circles (●). Environmental variables or selected fermentation products are represented by red arrows. Bacterial community at genus-like groups with the level, i.e. phylum (P), order (O), family (F) or genus (G) are represented as blue arrows. The arrows indicate the direction in which the relative abundance increases. Length of arrows is a measure of fit. The environmental variable arrows (in red) approximated the correlation between species and an environmental variable. The further a product falls in the direction indicated by an arrow, the higher the correlation. Both axes together explained 91.8% of the total variance in the dataset

The CANOCO (multivariate analysis) software programme was used to reveal the relation between the 16 most dominant bacteria (genus-like level) and the fermentation patterns in both reactors. First, PCA analysis (unweighted) was used to visualise the overall relationship of those in the Dutch and Thai reactors (Additional file 11: Figure S4). Then, the relationship between those top 16 bacteria and main products during the fermentation was analysed using RDA analysis and a correlation matrix (Spearman’s Rank Order Correlations statistics) (Figs. 4, 5; Additional file 12: Table S8).

In the Dutch reactor, the relationship between the bacterial composition and lactate, acetate and butyrate could be explained by first two canonical differentiation axes with 69% of all total datasets (*p* = 0.04) (Fig. 4). The results showed that in the first stage (day 0–1.33), the relative abundance of *Bacillus* and *Streptococcus* positively correlated with increasing lactate concentration (*r* = 0.860, *p* < 0.05 and *r* = 0.778, *p* < 0.05, respectively). In the second stage (day 1.33–2) (Additional file 12: Table S8a), lactate was consumed whilst butyrate was mainly produced. Mainly *Streptococcus* were present in this stage. The correlation results showed that *Parabacteroides* (*r* = 0.883, *p* < 0.01), *Sporanaerobacter* (*r* = 0.867, *p* < 0.05), Helicobacteraceae (*r* = 0.802, *p* < 0.05), Peptostreptococcaceae (*r* = 0.852, *p* < 0.05) and Porphyromonadaceae (*r* = 0.867, *p* < 0.05) positively correlated with butyrate production, whereas these members showed a negative correlation with lactate concentration (Figs. 1, 2, 4; Additional file 12: Table S8a). Moreover, members of Clostridiaceae, known as butyrate-producing bacteria, showed a significantly negative correlation with the lactate concentration (*r* = − 0.889, *p* < 0.01) and a positive correlation with the butyrate concentration (*r* = 0.607). In the late stage of the fermentation (day 4–10), acetate concentration increased and positively correlated with *Parabacteroides* (*r* = 0.775, *p* < 0.05) and this genus showed a positive correlation with butyrate as well (Fig. 4; Additional file 12: Table S8a). Members of the family Lachnospiraceae, known for their ability to convert lactate to butyrate or propionate [52], had a negative correlation (*r* = − 0.667) to lactate, but a positive correlation with butyrate (*r* = 0.393) and acetate (*r* = 0.286) in the Dutch reactor.

In the Thai reactor, the relationship between the bacterial composition and lactate, acetate, propionate and butyrate was explained by two canonical differentiation axes with 91.8% of all total data set (*p* = 0.01) (Fig. 5). The results revealed that in the first two stages: stage 1; 0–0.25, and stage 2; 0.25–3 (day 0–3), the relative abundance of genus *Streptococcus* increased in the same direction of increasing lactate. However, there was no significant correlation between bacterial community shift and lactate production (Additional file 12: Table S8b). In the late stage of the fermentation (day 4–16), a variety of bacteria were involved in acetate, butyrate and propionate formation (Fig. 5). Members of the genera *Bacteroides* (*r* = 0.964, *p* < 0.01), *Dysgonomonas* (*r* = 0.929, *p* < 0.01) and *Pseudoramibacter_Eubacterium* (*r* = 0.821, *p* < 0.05), and families Enterobacteriaceae (*r* = 0.857, *p* < 0.05) and Porphyromonadaceae (*r* = 0.821, *p* < 0.05) had significantly positive correlations with acetate, butyrate and propionate production (Additional file 12: Table S8b). Members of *Bacteroides* in general are known to produce acetate, succinate and propionate [53]. It was reported that the family Porphyromonadaceae, which includes the genus *Dysgonomonas*, possesses three butyrate synthesis pathways [54] and our findings support the relationship between this taxon and butyrate formation. *Eubacterium* spp. are known as butyrate-producing bacteria [52] and probably responsible for the conversion of lactate and acetate to butyrate. Remarkably, the Lachnospiraceae family had a negative correlation with lactate and a positive correlation with acetate and butyrate (*r* = 0.286 and *r* = 0.393, respectively) in the Dutch reactor but a positive correlation (*r* = 0.473) with lactate and a negative correlation with acetate, butyrate and propionate (*r* = − 0.929, *p* < 0.01) in the Thai reactor. In the Dutch reactor, Lachnospiraceae members may have been responsible for the conversion of lactate to acetate and/or butyrate. This is well known for bacteria from the *Clostridium* cluster XIVa group in the Lachnospiraceae family, which are acetate plus lactate-converting butyrate producers [55]. Besides, there are other Lachnospiraceae members such as *Eubacterium rectale* and *Roseburia inulinivorans* which are also known to produce butyrate, formate and lactate [56]. The other, *Eubacterium hallii*, consumed lactate and acetate and produced butyrate [56]. In the Thai reactor, *Pseudoramibacter_Eubacterium* had 18% relative abundance at day 4 and may also have been involved in butyrate formation from lactate plus acetate.

Lactate is produced by a broad range of microorganisms such as bacteria and/or fungi. Currently, available lactate-producing strains still have advantages and disadvantages, for instance, the fungus *Rhizopus oryzae* is used to commercially produce l(+) lactate because it can directly produce it from starch. However, its yield is lower compared to lactate produced by LAB and it has been reported that its mycelium caused turbidity and disturbed the reactor [2]. LAB produce lactate from glucose that mostly originate from corn syrup. As such, this feed stock competes with food and feed. Due to the increasing demand of lactate, further development of a lactate production platform is needed. As only a few LABs can meet the strict industrial requirements, such as capability to ferment low-cost materials rapidly, less requirement of nitrogenous nutrients, and high yields with small amounts of other by-products, there is a need for novel strains [2, 42].

Amylolytic lactic acid bacteria (ALAB) such as *Enterococcus faecium, E. durans, Lactobacillus* spp. and *Streptococcus* spp., which are capable of utilising starchy materials, are of biotechnological interest because of their potential to directly convert starchy biomass to lactate [57, 58]. This group (ALAB) produces lactate more effectively than LAB because they combine pre-treatment by enzymatic hydrolysis of carbohydrate and glucose fermentation to lactate in one step. In our study, various species of ALAB were detected and isolated from both reactors including *Enterococcus faecium*, *E. durans* and *Lactobacillus plantarum* (Additional file 6: Table S6). The *Streptococcus* members were the most successful due to their rapid and high lactate production from starch waste fermentation, increasing in relative abundances (from < 1% up to 86%) in both reactors. The majority of the isolates (D0, D0.25, D0.5, T0-3, T0.25, T0.5, T1 and T3-3) were *Streptococcus* members (Additional file 6: Table S6) and their 16S rRNA gene sequences showed 100% identity to the most abundant OTUs (pyrosequencing results), which play an important role in lactate production in both reactors. The BLASTN analysis of the 16S rRNA gene sequences of those isolates (ca. 1400 bp) showed ca. 99% identity to *Streptococcus lutetiensis,* a strain able to degrade starch [59, 60]. In 2013, Jiang et al. studied the fermentation of amylopectin and resistant starch (RS2) using colonic inocula of pigs and found that there was 4% relative abundance of *S. lutetiensis* detected from total lactic acid-producing bacteria in the early stage of the fermentation [61]. This result together with our findings suggests that *S. lutetiensis* plays an important role in starch waste fermentation in our reactors. In general, using a single LAB strain for lactate production from glucose has some disadvantages because it lacks several biosynthetic pathways and therefore requires addition of costly nitrogen sources (yeast extract and/or peptone) and sterile conditions [44]. In this aspect, starchy waste as substrate becomes an advantage from an economical point of view because it contains crude proteins and various sources needed for the ruminal microorganisms and/or ALAB.

Importantly, using a rumen-derived inoculum, high concentrations of lactate and other OAs can be reached from starch waste without the addition of hydrolytic enzymes. To produce other OA (acetate, butyrate and/or propionate), prolonged fermentation is required. Therefore, undefined mixed cultures originating from rumen are attractive for the production of organic acids, but specifically lactate, from starch waste.

## Conclusions

Our study confirms that the substrate (waste) composition and source of inoculum play important roles in OA production, and the microbial community development in anaerobic digestion is reflected by changes in product profile. Starch waste or starchy materials are an alternative source for lactate production and *Streptococcus* spp. are key microorganisms in this context. Using rumen fluid or isolated ALAB such as *Streptococcus* spp. in starch waste reactors to produce lactate is a promising approach. Different inoculum sources affected the secondary fermentation product profile. Rumen fluid is a suitable inoculum source because it contains various microorganisms with high capacity to convert organic waste to valuable products without the requirement of addition of hydrolytic enzymes. Due to the complexity of the rumen microbiota, it has also potential to produce products from other complex organic waste sources, such as kitchen waste and agricultural or industrial wastes containing cellulose and/or lignocellulosic materials.

## Additional files

**Additional file 1: Table S1.** Composition of the diets of Dutch and Thai cows.

**Additional file 2: Table S2.** Composition of the dried starch waste used in this study.

**Additional file 3: Table S3.** Production profiles of starch waste fermentation using the Dutch rumen fluid as inoculum.

**Additional file 4: Table S4.** Production profiles of starch waste fermentation using the Thai rumen fluid as inoculum.

**Additional file 5: Table S5.** Total bacterial counts (log_10_ CFU/ml) at different time point in during starch waste fermentation in the Dutch and Thai reactors.

**Additional file 6: Table S6.** Pure cultures isolated from the starch waste fermentation process. Ten different strains are in Bold.

**Additional file 7: Figure S1.** Heat map: The abundances of 16S rRNA reads from the Dutch and Thai reactors (Filter by count per OTU: 500 per taxon) at genus-like level were presented. The date of sampling was shown (days). The numbers in each cell represented number of reads in each sample and OTU IDs were shown in the last column.

**Additional file 8: Table S7.** The number of reads and OTUs per sample generated using 16S rRNA gene amplicon pyrosequencing from both reactors.

**Additional file 9: Figure S2.** Relative abundance (%) of bacterial communities in the starch waste fermentation at phylum level using the two different inoculum sources (Dutch and Thai rumen fluids). Phyla with an average relative abundance lower than 1% in all samples were grouped in the category others.

**Additional file 10: Figure S3.** Grouping tree of the bacterial communities from both reactors (a). PCA plot weighted unifraction of the relative abundance of the bacterial communities at different time points in the starch waste fermentation using the Dutch rumen fluid (red dots) and Thai rumen fluid (blue dots) (b).

**Additional file 11: Figure S4.** Principal component analysis (PCA) with unconstrained ordination triplot between the top 16 genus-like level phylogenetic groupings of the OTUs and the environmental variables explaining the variance with time in the Dutch (a) and Thai (b) reactors. Time points are indicated the sampling point (days) during the starch waste fermentation and shown as filled circles (●). Environmental variables or selected fermentation products are represented by red arrows. Bacterial community at genus-like groups with the level, i.e. phylum (P), order (O), family (F) or genus (G) are represented as blue arrows. The direction of the species, in which the species abundance increases. Length of arrows is a measure of fit. The environmental variable arrows (in red) approximated the correlation between species and an environmental variable. The further a product falls in the direction indicated by arrow, the higher the correlation. Both axes together explained 73 and 92.3% of the total variances in the datasets from the Dutch (a) and Thai (b) reactors, respectively.

**Additional file 12: Table S8.** Correlation matrix (Spearman’s Rank Order Correlations statistics) between Bacterial OTUs at genus-like level and the operational data from the Dutch reactor (a) and Thai reactor (b). Green colours indicate positive correlations, whereas red colours indicate negative correlations. Correlation is significant at the *p* = 0.05 level (2-tailed) for the groups in the solid parentheses, whereas the dashed parentheses indicate the significant correlations at *p* = 0.01 (2-tailed) and both font types are italic.

## Abbreviations

ALAB: amylolytic lactic acid bacteria
BM: bicarbonate-buffered anaerobic mineral medium
CFU: colony-forming units
DGGE: denaturing gradient gel electrophoresis
DNA: deoxyribonucleic acid
LAB: lactic acid bacteria
OA: organic acids
OTU: operational taxonomic units
PCA: principal component analysis
PCoA: principal coordinates analysis
PCR: polymerase chain reaction
PLA: polylactic acid
RDA: redundancy analysis
rRNA: ribosomal ribonucleic acid
VFA: volatile fatty acid
