# Supplementary material for: Organic acid production from potato starch waste fermentation by rumen microbial communities from Dutch and Thai dairy cows
Source: Biotechnol Biofuels. 2018 Jan 25;11:13. doi: 10.1186/s13068-018-1012-4 (PMC5784674; doi:10.1186/s13068-018-1012-4)
Supplement: Supplementary file 1 — Additional file 1: Table S1. Composition of the diets of Dutch and Thai cows. [file 13068_2018_1012_MOESM1_ESM.docx]

***Figures, Tables and Additional files for Dutch and Thai manuscript***

**Organic acid production in potato starch waste fermentation by rumen microbial communities from Dutch and Thai dairy cows**

Susakul Palakawong Na Ayudthaya^1, 2^, Antonius H.P. van de Weijer^1^, Antonie H. van Gelder^1^, Alfons J. M. Stams^1,3^, Willem M. de Vos^1,4^ and Caroline M. Plugge^1*^

^1^Laboratory of Microbiology, Wageningen University & Research, Stippeneng 4, 6708 WE Wageningen, The Netherlands

^2^Thailand Institute of Scientific and Technological Research, 35 Mu 3, Khlong Ha, Amphoe Khlong Luang, Pathum Thani 12120 Thailand

^3^CEB-Centre of Biological Engineering, University of Minho, Campus de Gualtar, 4710-057 Braga, Portugal

^4^RPU Immunology, Department of Bacteriology and Immunology, University of Helsinki, Haartmaninkatu 3, FIN-00014 Helsinki, Finland

*Correspondence: [caroline.plugge@wur.nl](mailto:susakul.palakawongnaayudthaya@wur.nl),

Tel. + 31 (0) 317 483752

**Additional file 1: Table S1.** Composition of the diets of Dutch and Thai cows

*Location of the cows*

**Dutch cow:** Carus house, Department of Animal Sciences, Bornse Weilanden 5, Wageningen University, Wageningen, 6708 WG The Netherlands

**Thai cow:** CP Test Farm Highway 3138 Road, Tambon Khlong Kio, Amphoe Ban Bueng, Chon Buri 20220, Thailand

| Feed composition | | Dry matter intake per cow per day (kg) |
| --- | --- | --- |
| *Dutch cow*  Pelleted feed (20% protein; 25% starch)  Maize silage (7-7.5% protein; 35-40% starch)  Grass silage (15% protein; 0% starch) | | 7.0  6.5  6.5 |
| *Thai cow*  Pelleted feed (14% protein; 30% starch)  Pineapple peel (0% starch) | 3.52 – 4.40  3.25 | |
