# Supplementary material for: Organic acid production from potato starch waste fermentation by rumen microbial communities from Dutch and Thai dairy cows
Source: Biotechnol Biofuels. 2018 Jan 25;11:13. doi: 10.1186/s13068-018-1012-4 (PMC5784674; doi:10.1186/s13068-018-1012-4)
Supplement: Supplementary file 2 — Additional file 2: Table S2. Composition of the dried starch waste used in this study. [file 13068_2018_1012_MOESM2_ESM.docx]

***Figures, Tables and Additional files for Dutch and Thai manuscript***

**Organic acid production in potato starch waste fermentation by rumen microbial communities from Dutch and Thai dairy cows**

Susakul Palakawong Na Ayudthaya^1, 2^, Antonius H.P. van de Weijer^1^, Antonie H. van Gelder^1^, Alfons J. M. Stams^1,3^, Willem M. de Vos^1,4^ and Caroline M. Plugge^1*^

^1^Laboratory of Microbiology, Wageningen University & Research, Stippeneng 4, 6708 WE Wageningen, The Netherlands

^2^Thailand Institute of Scientific and Technological Research, 35 Mu 3, Khlong Ha, Amphoe Khlong Luang, Pathum Thani 12120 Thailand

^3^CEB-Centre of Biological Engineering, University of Minho, Campus de Gualtar, 4710-057 Braga, Portugal

^4^RPU Immunology, Department of Bacteriology and Immunology, University of Helsinki, Haartmaninkatu 3, FIN-00014 Helsinki, Finland

*Correspondence: [caroline.plugge@wur.nl](mailto:susakul.palakawongnaayudthaya@wur.nl),

Tel. + 31 (0) 317 483752

**Additional file 2: Table S2.** Composition of the dried starch waste used in this study

Starch waste was obtained from Avebe Potato factory (Foxhol, The Netherlands) and the starch waste composition was analysed at Nutricontrol (Veghel, The Netherlands)

| Composition | Percent (% w w^-1^) |
| --- | --- |
| Starch  Crude protein  Fibre  Crude ash  Sugar  Fat  Others | 61  1.1  0.8  0.4  0.3  0.01  36.39 |
