# Supplementary material for: Organic acid production from potato starch waste fermentation by rumen microbial communities from Dutch and Thai dairy cows
Source: Biotechnol Biofuels. 2018 Jan 25;11:13. doi: 10.1186/s13068-018-1012-4 (PMC5784674; doi:10.1186/s13068-018-1012-4)
Supplement: Supplementary file 3 — Additional file 3: Table S3. Production profiles of starch waste fermentation using the Dutch rumen fluid as inoculum. [file 13068_2018_1012_MOESM3_ESM.docx]

***Figures, Tables and Additional files for Dutch and Thai manuscript***

**Organic acid production in potato starch waste fermentation by rumen microbial communities from Dutch and Thai dairy cows**

Susakul Palakawong Na Ayudthaya^1, 2^, Antonius H.P. van de Weijer^1^, Antonie H. van Gelder^1^, Alfons J. M. Stams^1,3^, Willem M. de Vos^1,4^ and Caroline M. Plugge^1*^

^1^Laboratory of Microbiology, Wageningen University & Research, Stippeneng 4, 6708 WE Wageningen, The Netherlands

^2^Thailand Institute of Scientific and Technological Research, 35 Mu 3, Khlong Ha, Amphoe Khlong Luang, Pathum Thani 12120 Thailand

^3^CEB-Centre of Biological Engineering, University of Minho, Campus de Gualtar, 4710-057 Braga, Portugal

^4^RPU Immunology, Department of Bacteriology and Immunology, University of Helsinki, Haartmaninkatu 3, FIN-00014 Helsinki, Finland

*Correspondence: [caroline.plugge@wur.nl](mailto:susakul.palakawongnaayudthaya@wur.nl),

Tel. + 31 (0) 317 483752

**Additional file 3: Table S3**. Production profiles of starch waste fermentation using the Dutch rumen fluid as inoculum

| **Sample (Days)** | Glucose | **VFAs/OAs (mmol l^-1^)** | | | | | | | Ethanol | |  |
| --- | --- | --- | --- | --- | --- | --- | --- | --- | --- | --- | --- |
|  | (mmol l^-1^) | *Lactate* | *Formate* | *Succinate* | *Acetate* | *Propionate* | *Butyrate* | *Iso-valerate* | | (mmol l^-1^) |  |
| *Substrate* | 0.3 (+0.02) | 17.7 (+0.99) | 0 | 0.6 (+0.15) | 6.4 (+0.70) | 2.5 (+0.25) | 0 | 0 | | 6.6 (+0.53) | |
| *0* | 0 | 18.7 (+0.89) | 0 | 0.6 (+0.14) | 7.6 (+0.62) | 3.1 (+0.46) | 1.1 (+0.05) | 0 | | 6.7 (+0.55) | |
| *0.25* | 0 | 33.2 (+0.80) | 5.2 (+0.59) | 1.1 (+0.02) | 11.5 (+0.27) | 2.8 (+0.09) | 0.9 (+0.05) | 0 | | 8.6 (+0.23) | |
| *0.5* | 0.2 (+0.02) | 55.3 (+1.03) | 18.6 (+0.65) | 1.1 (+0.02) | 22.1 (+0.28) | 2.5 (+0.11) | 2.7 (+0.04) | 0 | | 23.4 (+0.10) | |
| *1* | 0.3 (+0.01) | 140.1 (+4.39) | 22.4 (+0.29) | 1.8 (+0.08) | 82.1 (+2.40) | 6.2 (+0.12) | 3.7 (+0.08) | 0 | | 60.9 (+0.54) | |
| 1.3 | 0 | 147.7 (+3.19) | 44.3 (+0.13) | 1.7 (+0.12) | 93.3 (+1.97) | 6.0 (+0.11) | 4.3 (+0.15) | 0.6 (+0.04) | | 72.8 (+0.52) | |
| *2* | 0 | 12.7 (+0.15) | 57.7 (+1.16) | 3.4 (+0.05) | 46.4 (+1.59) | 5.0 (+0.06) | 112.1 (+2.01) | 1.1 (+0.02) | | 74.6 (+0.26) | |
| *3* | 0 | 0 | 63.5 (+1.96) | 5.2 (+0.11) | 51.7 (+1.29) | 5.7 (+0.01) | 126.4 (+2.96) | 1.6 (+0.03) | | 70.2 (+0.17) | |
| *4* | 0 | 0 | 61.7 (+3.40) | 4.5 (+0.21) | 61.2 (+2.66) | 7.9 (+0.11) | 125.5 (+5.97) | 2.0 (+0.04) | | 63.5 (+1.87) | |
| *5* | 0 | 0 | 53.0 (+1.42) | 0 | 70.2 (+1.95) | 11.7 (+0.19) | 124.8 (+3.54) | 2.6 (+0.00) | | 59.2 (+0.61) | |
| *6* | 0 | 0 | 40.2 (+1.23) | 0 | 79.7 (+2.67) | 12.6 (+0.22) | 125.4 (+4.34) | 2.8 (+0.01) | | 55.1 (+0.72) | |
| *7* | 0 | 0 | 20.4 (+0.48) | 0 | 89.0 (+2.66) | 13.2 (+0.18) | 127.0 (+4.39) | 3.0 (+0.03) | | 52.1 (+0.69) | |
| *8* | 0 | 0 | 9.8 (+0.19) | 0 | 102.7 (+1.55) | 14.2 (+0.27) | 138.4 (+2.09) | 3.3 (+0.03) | | 52.9 (+0.73) | |
| *10* | 0 | 0 | 0 | 0 | 115.1 (+1.41) | 15.4 (+0.16) | 145.5 (+2.20) | 3.6 (+0.06) | | 52.3 (+0.84) | |
| *11* | 0 | 0 | 0 | 0 | 118.1 (+2.30) | 15.6 (+0.34) | 146.0 (+2.96) | 3.6 (+0.09) | | 50.5 (+0.71) | |
| *12* | 0 | 0 | 0 | 0 | 119.6 (+1.69) | 15.8 (+0.19) | 144.7 (+2.18) | 3.6 (+0.07) | | 48.2 (+1.02) | |
| *13* | 0 | 0 | 0 | 0 | 124.4 (+4.47) | 16.2 (+0.55) | 147.4 (+5.42) | 3.6 (+0.16) | | 46.0 (+1.35) | |
| *14* | 0 | 0 | 0 | 0 | 131.2 (+3.92) | 16.9 (+0.51) | 152.6 (+4.60) | 3.6 (+0.10) | | 44.5 (+1.15) | |
| *15* | 0 | 0 | 0 | 0 | 135.0 (+1.49) | 17.3 (+0.18) | 152.3 (+1.80) | 3.7 (+0.04) | | 42.2 (+0.25) | |
| *16* | 0 | 0 | 0 | 0 | 124.0 (+1.61) | 15.6 (+0.21) | 137.0 (+2.12) | 3.2 (+0.06) | | 35.2 (+0.49) | |

All values are averages of technical duplicates and red values were the highest amount of each product. Values in parentheses are standard deviations.
