# Supplementary material for: Organic acid production from potato starch waste fermentation by rumen microbial communities from Dutch and Thai dairy cows
Source: Biotechnol Biofuels. 2018 Jan 25;11:13. doi: 10.1186/s13068-018-1012-4 (PMC5784674; doi:10.1186/s13068-018-1012-4)
Supplement: Supplementary file 4 — Additional file 4: Table S4. Production profiles of starch waste fermentation using the Thai rumen fluid as inoculum. [file 13068_2018_1012_MOESM4_ESM.docx]

***Figures, Tables and Additional files for Dutch and Thai manuscript***

**Organic acid production in potato starch waste fermentation by rumen microbial communities from Dutch and Thai dairy cows**

Susakul Palakawong Na Ayudthaya^1, 2^, Antonius H.P. van de Weijer^1^, Antonie H. van Gelder^1^, Alfons J. M. Stams^1,3^, Willem M. de Vos^1,4^ and Caroline M. Plugge^1*^

^1^Laboratory of Microbiology, Wageningen University & Research, Stippeneng 4, 6708 WE Wageningen, The Netherlands

^2^Thailand Institute of Scientific and Technological Research, 35 Mu 3, Khlong Ha, Amphoe Khlong Luang, Pathum Thani 12120 Thailand

^3^CEB-Centre of Biological Engineering, University of Minho, Campus de Gualtar, 4710-057 Braga, Portugal

^4^RPU Immunology, Department of Bacteriology and Immunology, University of Helsinki, Haartmaninkatu 3, FIN-00014 Helsinki, Finland

*Correspondence: [caroline.plugge@wur.nl](mailto:susakul.palakawongnaayudthaya@wur.nl),

Tel. + 31 (0) 317 483752

**Additional file 4: Table S4**. Production profiles of starch waste fermentation using the Thai rumen fluid as inoculum

| **Sample (Days)** | Glucose | **VFAs/OAs (mmol l^-1^)** | | | | | | | | | Ethanol |  |
| --- | --- | --- | --- | --- | --- | --- | --- | --- | --- | --- | --- | --- |
|  | (mmol l^-1^) | *Lactate* | *Formate* | *Succinate* | *Acetate* | *Propionate* | *Butyrate* | *Valerate* | *Iso-valerate* | (mmol l^-1^) | | |
| *Substrate* | 0.5 (+0.02) | 17.1 (+0.2) | 0 | 0.7 (+0.03) | 5.9 (+0.04) | 2.5 (+0.2) | 0.8 (+0.04) | 0 | 0 | 6.6 (+0.5) | | |
| *0* | 0.5 (+0.02) | 17.0 (+0.2) | 0 | 0.8 (+0.00) | 6.8 (+0.2) | 2.9 (+0.09) | 1.2 (+0.08) | 0 | 0 | 6.6 (+0.3) | | |
| *0.25* | 0 | 19.2 (+0.5) | 0 | 0.7 (+0.09) | 7.3 (+0.1) | 2.9 (+0.3) | 1.1 (+0.03) | 0 | 0 | 6.8 (+0.04) | | |
| *0.5* | 1.5 (+1.9) | 244.6 (+6.0) | 6.8 (+0.5) | 0 | 9.3 (+0.4) | 5.5 (+0.2) | 1.1 (+0.1) | 0 | 0 | 8.4 (+0.5) | | |
| *1* | 0 | 266.3 (+8.6) | 32.3 (+1.4) | 1.0 (+0.2) | 24.0 (+1.2) | 5.3 (+0.3) | 4.5 (+0.2) | 0 | 0 | 26.0 (+2.0) | | |
| 1.3 | 0 | 251.0 (+1.5) | 24.4 (+0.4) | 1.3 (+0.4) | 30.3 (+0.04) | 5.6 (+0.5) | 4.8 (+0.2) | 0 | 0 | 29.3 (+1.2) | | |
| *2* | 0 | 257.7 (+2.5) | 11.2 (+0.4) | 1.5 (+0.4) | 38.3 (+1.2) | 5.5 (+0.05) | 6.3 (+0.8) | 0 | 0 | 28.8 (+6.9) | | |
| *3* | 0 | 231.4 (+10.3) | 0 | 1.5 (+0.4) | 48.0 (+1.9) | 18.0 (+0.7) | 14.1 (+0.4) | 0 | 0 | 35.7 (+0.02) | | |
| *4* | 0 | 0 | 0 | 0 | 147.2 (+9.1) | 102.2 (+8.0) | 63.0 (+4.2) | 12.3 (+0.05) | 1.1 (+0.05) | 26.2 (+1.6) | | |
| *5* | 0 | 0 | 0 | 0 | 150.7 (+0.7) | 61.4 (+0.5) | 61.4 (+0.5) | 12.4 (+0.2) | 1.4 (+0.03) | 21.5 (+0.9) | | |
| *6* | 0 | 0 | 0 | 0 | 159.8 (+8.7) | 104.8 (+5.8) | 62.1 (+3.7) | 12.9 (+0.7) | 1.9 (+0.1) | 18.9 (+1.6) | | |
| *7* | 0 | 0 | 0 | 0 | 167.8 (+12.5) | 106.6 (+8.3) | 63.8 (+4.9) | 13.1 (+1.0) | 2.3 (+0.1) | 19.8 (+1.4) | | |
| *8* | 0 | 0 | 0 | 0 | 189.0 (+0.5) | 118.7 (+0.2) | 70.6 (+0.01) | 14.5 (+0.02) | 2.6 (+0.01) | 21.4 (+0.01) | | |
| *10* | 0 | 0 | 0 | 0 | 190.6 (+3.4) | 116.6 (+2.6) | 69.0 (+1.5) | 14.0 (+0.3) | 2.6 (+0.09) | 18.4 (+0.7) | | |
| *11* | 0 | 0 | 0 | 0 | 183.5 (+7.9) | 111.0 (+5.0) | 65.2 (+2.9) | 13.4 (+0.6) | 2.5 (+0.08) | 16.2 (+1.0) | | |
| *12* | 0 | 0 | 0 | 0 | 188.6 (+3.0) | 112.8 (+1.9) | 65.9 (+1.0) | 13.6 (+0.2) | 2.5 (+0.00) | 15.3 (+0.3) | | |
| *13* | 0 | 0 | 0 | 0 | 192.2 (+8.3) | 114.9 (+5.2) | 66.7 (+3.0) | 13.9 (+0.6) | 2.6 (+0.2) | 14.7 (+0.9) | | |
| *14* | 0 | 0 | 0 | 0 | 193.9 (+4.6) | 117.6 (+3.2) | 68.1 (+1.9) | 14.1 (+0.4) | 2.6 (+0.05) | 13.7 (+0.7) | | |
| *15* | 0 | 0 | 0 | 0 | 183.9 (+3.3) | 118.0 (+2.1) | 68.1 (+1.1) | 14.2 (+0.2) | 2.5 (+0.06) | 12.8 (+0.1) | | |
| *16* | 0 | 0 | 0 | 0 | 200.4 (+3.3) | 148.8 (+2.4) | 85.9 (+1.4) | 18.1 (+0.4) | 3.8 (+0.04) | 14.8 (+0.3) | | |

All values are averages of technical duplicates and red values were the highest amount of each product. Values in parentheses are standard deviations.
