# Supplementary material for: Organic acid production from potato starch waste fermentation by rumen microbial communities from Dutch and Thai dairy cows
Source: Biotechnol Biofuels. 2018 Jan 25;11:13. doi: 10.1186/s13068-018-1012-4 (PMC5784674; doi:10.1186/s13068-018-1012-4)
Supplement: Supplementary file 5 — Additional file 5: Table S5. Total bacterial counts (log10 CFU/ml) at different time point in during starch waste fermentation in the Dutch and Thai reactors. [file 13068_2018_1012_MOESM5_ESM.docx]

***Figures, Tables and Additional files for Dutch and Thai manuscript***

**Organic acid production in potato starch waste fermentation by rumen microbial communities from Dutch and Thai dairy cows**

Susakul Palakawong Na Ayudthaya^1, 2^, Antonius H.P. van de Weijer^1^, Antonie H. van Gelder^1^, Alfons J. M. Stams^1,3^, Willem M. de Vos^1,4^ and Caroline M. Plugge^1*^

^1^Laboratory of Microbiology, Wageningen University & Research, Stippeneng 4, 6708 WE Wageningen, The Netherlands

^2^Thailand Institute of Scientific and Technological Research, 35 Mu 3, Khlong Ha, Amphoe Khlong Luang, Pathum Thani 12120 Thailand

^3^CEB-Centre of Biological Engineering, University of Minho, Campus de Gualtar, 4710-057 Braga, Portugal

^4^RPU Immunology, Department of Bacteriology and Immunology, University of Helsinki, Haartmaninkatu 3, FIN-00014 Helsinki, Finland

*Correspondence: [caroline.plugge@wur.nl](mailto:susakul.palakawongnaayudthaya@wur.nl),

Tel. + 31 (0) 317 483752

**Additional file 5: Table S5.**

**Total bacterial counts** (log_10_CFU/ml) at different time point in during starch waste fermentation in the Dutch and Thai reactors

| **Bioreactor** | **Log_10_ CFU/ml (standard deviation: SD) at different time points**  **Days** | | | | | | | | | | | | | |  |
| --- | --- | --- | --- | --- | --- | --- | --- | --- | --- | --- | --- | --- | --- | --- | --- |
|  | **0** | **0.25** | **0.5** | **1** | **2** | **3** | **4** | **5** | **6** | **7** | **8** | **10** | **14** | **16** | |
| **Dutch** | 7.9 (+ 0.3) | 7.1 (+ 0.1) | 7.4 (+ 0.0) | 10.3 (+ 0.0) | 8.9 (+ 0.1) | 9.3 (+ 0.1) | 7.0 (+ 0.0) | 6.7 (+ 0.2) | 7.2 (+ 0.0) | (7.1 + 0.1) | 6.7 (+ 0.1) | 7.3 (+ 0.1) | 6.1 (+ 0.0) | 4.8 (+ 0.0) | |
| **Thai** | 7.9 (+ 0.1) | 8.3 (+ 0.1) | 10.3 (+ 0.1) | 10.3 (+ 0.1) | 10.1 (+ 0.1) | 9.4 (+ 0.0) | 9.1 (+ 0.1) | 6.7 (+ 0.2) | 8.6 (+ 0.1) | (8.4 + 0.1) | 8.1 (+ 0.1) | 7.0 (+ 0.1) | 6.4 (+ 0.0) | 6.4 (+ 0.0) | |

All values are averages of technical triplicates and red values were the highest amount of each reactor. Values in parenthesises are standard deviations.
