# Supplementary material for: Organic acid production from potato starch waste fermentation by rumen microbial communities from Dutch and Thai dairy cows
Source: Biotechnol Biofuels. 2018 Jan 25;11:13. doi: 10.1186/s13068-018-1012-4 (PMC5784674; doi:10.1186/s13068-018-1012-4)
Supplement: Supplementary file 6 — Additional file 6: Table S6. Pure cultures isolated from the starch waste fermentation process. Ten different strains are in Bold. [file 13068_2018_1012_MOESM6_ESM.docx]

***Figures, Tables and Additional files for Dutch and Thai manuscript***

**Organic acid production in potato starch waste fermentation by rumen microbial communities from Dutch and Thai dairy cows**

Susakul Palakawong Na Ayudthaya^1, 2^, Antonius H.P. van de Weijer^1^, Antonie H. van Gelder^1^, Alfons J. M. Stams^1,3^, Willem M. de Vos^1,4^ and Caroline M. Plugge^1*^

^1^Laboratory of Microbiology, Wageningen University & Research, Stippeneng 4, 6708 WE Wageningen, The Netherlands

^2^Thailand Institute of Scientific and Technological Research, 35 Mu 3, Khlong Ha, Amphoe Khlong Luang, Pathum Thani 12120 Thailand

^3^CEB-Centre of Biological Engineering, University of Minho, Campus de Gualtar, 4710-057 Braga, Portugal

^4^RPU Immunology, Department of Bacteriology and Immunology, University of Helsinki, Haartmaninkatu 3, FIN-00014 Helsinki, Finland

*Correspondence: [caroline.plugge@wur.nl](mailto:susakul.palakawongnaayudthaya@wur.nl),

Tel. + 31 (0) 317 483752

**Additional file 6: Table S6.** Pure cultures isolated from the starch waste fermentation process. Ten different strains are in **Bold.**

| **Reactor** | **Sampling time (Days)** | **Sample name** | **Closest culture relative** | **Gram reaction** | **Identity (%)** | **Nº isolate** | **Acc. number of isolate** | **Sequence length** |
| --- | --- | --- | --- | --- | --- | --- | --- | --- |
|  | **0** | D0 | ***Streptococcus equinus*** | **+** | **99** | **1** | MF581503 | **1422** |
|  | 0.25 | D0.25 | *Streptococcus equinus* | + | 99 | 1 | MF581504 | 1422 |
| **Dutch** | **0=0.25=0.5** | D0, 0.25, 0.5 | ***Streptococcus lutetiensis*** | **+** | **98** | **1** | MF581505 | **1221** |
|  | **0.5** | D0.5 | ***Enterococcus faecium*** | **+** | **99** | **1** | MF581506 | **1440** |
|  | 1 | D1.1 | *Enterococcus faecium* | + | 99 | 1 | MF581507 | 1429 |
|  | 1 | D1.2 | *Enterococcus faecium* | + | 98 | 1 | MF581508 | 1456 |
|  | **8=10** | D8, 10 | ***Enterococcus gallinarum*** | **+** | **99** | **1** | MF581509 | **1435** |
|  | 9 | D9-1 | *Enterococcus faecium* | + | 99 | 1 | MF581510 | 1440 |
|  | **9** | D9-2 | ***Escherichia fergusonii*** | **-** | **99** | **1** | MF581511 | **1415** |
|  | **0** | T0-1 | ***Lactobacillus ghanensis*** | **+** | **99** | **1** | MF581512 | **1443** |
| **Thai** | 0 | T0-2 | *Enterococcus faecium* | + | 99 | 1 | MF581513 | 1435 |
|  | 0 | T0-3 | *Streptococcus lutetiensis* | + | 98 | 1 | MF581514 | 1430 |
|  | **0=0.25=0.5** | T0, 0.25, 0.5 | ***Lactobacillus plantarum*** | **+** | **99** | **1** | MF581515 | **1443** |
|  | 0.25 | T0.25 | *Streptococcus lutetiensis* | + | 99 | 1 | MF581516 | 1418 |
|  | 0.5 | T0.5 | *Streptococcus lutetiensis* | + | 99 | 1 | MF581517 | 1423 |
|  | 1 | T1 | *Streptococcus lutetiensis* | + | 99 | 1 | MF581518 | 1419 |
|  | 2 | T2-1 | *Enterococcus faecium* | + | 99 | 1 | MF581519 | 1428 |
|  | 2 | T2-2 | *Enterococcus gallinarum* | + | 99 | 1 | MF581520 | 1430 |
|  | **3** | T3-1 | ***Enterococcus durans*** | **+** | **97** | **1** | MF581521 | **1205** |
|  | 3 | T3-2 | *Enterococcus gallinarum* | + | 99 | 1 | MF581522 | 1425 |
|  | 3 | T3-3 | *Streptococcus lutetiensis* | + | 99 | 1 | MF581523 | 1428 |
|  | 8=10 | T8, 10-1 | *Enterococcus faecium* | + | 98 | 1 | MF581524 | 1443 |
|  | 8=10 | T8, 10-2 | *Enterococcus gallinarum* | + | 99 | 1 | MF581525 | 1428 |
|  | **12** | T12-1 | ***Clostridium sporogenes*** | **+** | **99** | **1** | MF581526 | **1384** |
|  | 12 | T12-2 | *Enterococcus faecium* | + | 99 | 1 | MF581527 | 1434 |
|  | 12 | T12-3 | *Enterococcus faecium* | + | 99 | 1 | MF581528 | 1417 |
|  | **12** | T12-4 | ***Eubacterium limosum*** | **+** | **99** | **1** | MF581529 | **1403** |
|  | 12 | T12-5 | *Lactobacillus ghanensis* | + | 99 | 1 | MF581530 | 1444 |
