# Supplementary material for: Organic acid production from potato starch waste fermentation by rumen microbial communities from Dutch and Thai dairy cows
Source: Biotechnol Biofuels. 2018 Jan 25;11:13. doi: 10.1186/s13068-018-1012-4 (PMC5784674; doi:10.1186/s13068-018-1012-4)
Supplement: Supplementary file 7 — Additional file 7: Figure S1. Heat map: The abundances of 16S rRNA reads from the Dutch and Thai reactors (Filter by count per OTU: 500 per taxon) at genus-like level were presented. The date of sampling was shown (days). The numbers in each cell represented number of reads in each sample and OTU IDs were shown in the last column. [file 13068_2018_1012_MOESM7_ESM.docx]

***Figures, Tables and Additional files for Dutch and Thai manuscript***

**Organic acid production in potato starch waste fermentation by rumen microbial communities from Dutch and Thai dairy cows**

Susakul Palakawong Na Ayudthaya^1, 2^, Antonius H.P. van de Weijer^1^, Antonie H. van Gelder^1^, Alfons J. M. Stams^1,3^, Willem M. de Vos^1,4^ and Caroline M. Plugge^1*^

^1^Laboratory of Microbiology, Wageningen University & Research, Stippeneng 4, 6708 WE Wageningen, The Netherlands

^2^Thailand Institute of Scientific and Technological Research, 35 Mu 3, Khlong Ha, Amphoe Khlong Luang, Pathum Thani 12120 Thailand

^3^CEB-Centre of Biological Engineering, University of Minho, Campus de Gualtar, 4710-057 Braga, Portugal

^4^RPU Immunology, Department of Bacteriology and Immunology, University of Helsinki, Haartmaninkatu 3, FIN-00014 Helsinki, Finland

*Correspondence: [caroline.plugge@wur.nl](mailto:susakul.palakawongnaayudthaya@wur.nl),

Tel. + 31 (0) 317 483752

| **Consensus Lineage** | **Dutch reactor** | | | | | | |  | **Thai reactor** | | | | | | | **#OTU ID** |
| --- | --- | --- | --- | --- | --- | --- | --- | --- | --- | --- | --- | --- | --- | --- | --- | --- |
|  | **Inoculum** | **0.25** | **0.5** | **1.33** | **2** | **4** | **10** |  | **Inoculum** | **0** | **2** | **3** | **4** | **7** | **16** |  |
| ***k__Bacteria; p__Bacteroidetes; c__Bacteroidia; o__Bacteroidales; f__Bacteroidaceae; g__Bacteroides*** |  |  |  |  |  |  |  |  | **9** | **2** | **138** | **1740** | **2432** | **2857** | **2840** | **denovo3672** |
| ***k__Bacteria; p__Bacteroidetes; c__Bacteroidia; o__Bacteroidales; f__Porphyromonadaceae*** |  |  |  |  | **19** | **10112** | **2031** |  |  |  |  |  |  |  |  | **denovo3391** |
| ***k__Bacteria; p__Bacteroidetes; c__Bacteroidia; o__Bacteroidales; f__Porphyromonadaceae*** |  |  |  |  |  |  | **921** |  | **31** | **3** | **57** | **6** | **89** | **682** | **3619** | **denovo3662** |
| ***k__Bacteria; p__Bacteroidetes; c__Bacteroidia; o__Bacteroidales; f__Porphyromonadaceae; g__Dysgonomonas*** |  |  |  |  |  |  |  |  | **4** |  | **24** | **227** | **551** | **852** | **514** | **denovo330** |
| ***k__Bacteria; p__Bacteroidetes; c__Bacteroidia; o__Bacteroidales; f__Porphyromonadaceae; g__Parabacteroides*** |  |  |  | **2** | **3** | **764** | **3432** |  |  |  |  |  |  |  |  | **denovo414** |
| ***k__Bacteria; p__Bacteroidetes; c__Bacteroidia; o__Bacteroidales; f__Prevotellaceae; g__Prevotella*** | **183** | **92** | **54** | **14** | **9** | **3** |  |  | **84** | **166** | **1** |  |  |  |  | **denovo2513** |
| ***k__Bacteria; p__Bacteroidetes; c__Bacteroidia; o__Bacteroidales; f__Prevotellaceae; g__Prevotella*** | **237** | **158** | **45** | **25** | **20** | **3** | **1** |  | **14** | **32** |  |  |  |  |  | **denovo3238** |
| ***k__Bacteria; p__Bacteroidetes; c__Bacteroidia; o__Bacteroidales; f__Prevotellaceae; g__Prevotella*** | **396** | **89** | **32** | **24** | **25** | **4** |  |  | **5** | **6** |  |  |  |  |  | **denovo4048** |
| ***k__Bacteria; p__Bacteroidetes; c__Bacteroidia; o__Bacteroidales; f__Prevotellaceae; g__Prevotella*** | **90** | **49** | **16** | **12** | **4** | **3** |  |  | **384** | **347** |  |  |  |  |  | **denovo4264** |
| ***k__Bacteria; p__Bacteroidetes; c__Bacteroidia; o__Bacteroidales; f__Prevotellaceae; g__Prevotella*** | **340** | **95** | **35** | **26** | **22** | **2** | **1** |  | **74** | **161** |  |  |  |  |  | **denovo5804** |
| ***k__Bacteria; p__Firmicutes; c__Bacilli; o__Lactobacillales*** |  |  |  |  |  |  |  |  | **64** | **446** | **2** | **2** | **3** |  | **8** | **denovo2459** |
| ***k__Bacteria; p__Firmicutes; c__Bacilli; o__Bacillales; f__Bacillaceae; g__Bacillus*** |  | **2302** | **27** | **14** | **13** | **15** |  |  |  |  |  |  |  |  |  | **denovo5113** |
| ***k__Bacteria; p__Firmicutes; c__Bacilli; o__Bacillales; f__Paenibacillaceae; g__Paenibacillus*** |  | **6867** | **264** | **19** | **1** | **2** |  |  |  |  |  |  |  |  |  | **denovo1660** |
| ***k__Bacteria; p__Firmicutes; c__Bacilli; o__Lactobacillales; f__Lactobacillaceae; g__Lactobacillus; s__zeae*** |  |  |  |  |  |  |  |  | **969** | **1183** | **18** | **21** | **34** | **20** | **35** | **denovo1761** |
| ***k__Bacteria; p__Firmicutes; c__Bacilli; o__Lactobacillales; f__Enterococcaceae; g__Enterococcus; s__cecorum*** |  |  |  |  |  |  |  |  |  |  | **242** | **226** | **31** | **10** |  | **denovo2047** |
| ***k__Bacteria; p__Firmicutes; c__Bacilli; o__Lactobacillales; f__Enterococcaceae; g__Enterococcus; s__casseliflavus*** |  |  |  |  |  |  |  |  | **5** |  | **59** | **54** | **73** | **105** | **422** | **denovo2206** |
| ***k__Bacteria; p__Firmicutes; c__Bacilli; o__Lactobacillales; f__Streptococcaceae; g__Streptococcus*** | **4** | **3656** | **19712** | **13003** | **14731** | **7260** | **1291** |  | **20** | **5** | **13251** | **10157** | **4845** | **1995** | **175** | **denovo4866** |
| ***k__Bacteria; p__Firmicutes; c__Clostridia; o__Clostridiales; f__Clostridiaceae*** |  |  |  |  | **19** | **3686** | **179** |  |  |  |  |  |  |  |  | **denovo730** |
| ***k__Bacteria; p__Firmicutes; c__Clostridia; o__Clostridiales; f__Clostridiaceae; g__Clostridium*** | **1** |  | **36** | **102** | **442** | **112** | **6** |  |  |  |  |  |  |  |  | **denovo5724** |
| ***k__Bacteria; p__Firmicutes; c__Clostridia; o__Clostridiales; f__Clostridiaceae; g__Clostridium*** |  | **7** | **411** | **32** | **16** | **61** | **6** |  |  |  |  |  |  |  |  | **denovo5763** |
| ***k__Bacteria; p__Firmicutes; c__Clostridia; o__Clostridiales; f__Clostridiaceae; g__Clostridium*** |  |  | **24** | **1216** | **1768** | **243** | **13** |  |  |  |  |  |  |  |  | **denovo2222** |
| ***k__Bacteria; p__Firmicutes; c__Clostridia; o__Clostridiales; f__Clostridiaceae; g__Clostridium; s__thermopalmarium*** |  |  | **440** | **24** | **1215** | **1675** | **34** |  |  |  |  |  |  |  |  | **denovo2069** |
| ***k__Bacteria; p__Firmicutes; c__Clostridia; o__Clostridiales; f__Eubacteriaceae; g__Pseudoramibacter_Eubacterium*** |  |  |  |  |  |  |  |  |  |  | **10** | **24** | **1686** | **1224** | **594** | **denovo311** |
| ***k__Bacteria; p__Firmicutes; c__Clostridia; o__Clostridiales; f__Eubacteriaceae; g__Pseudoramibacter_Eubacterium*** |  |  |  |  |  |  |  |  |  |  | **9** | **3** | **592** | **317** | **382** | **denovo375** |
| ***k__Bacteria; p__Firmicutes; c__Clostridia; o__Clostridiales; f__Peptostreptococcaceae*** |  |  |  | **1** | **2015** | **325** | **402** |  |  |  |  |  | **16** | **21** | **6** | **denovo2839** |
| ***k__Bacteria; p__Firmicutes; c__Clostridia; o__Clostridiales; f__[Tissierellaceae]; g__Sporanaerobacter*** |  |  |  |  | **2** | **1514** | **435** |  |  |  |  |  |  |  |  | **denovo1590** |
| ***k__Bacteria; p__Firmicutes; c__Clostridia; o__Clostridiales; f__[Tissierellaceae]; g__Peptoniphilus*** |  |  |  |  |  |  |  |  |  |  | **4** |  |  | **327** | **229** | **denovo3616** |
| ***k__Bacteria; p__Firmicutes; c__Clostridia; o__Clostridiales; f__Ruminococcaceae; g__Ruminococcus*** |  |  |  |  |  |  |  |  | **3** |  | **2** |  |  |  | **1176** | **denovo3772** |
| ***k__Bacteria; p__Firmicutes; c__Clostridia; o__Clostridiales; f__Veillonellaceae*** |  |  |  |  |  |  |  |  |  |  | **2** | **19** | **406** | **255** | **62** | **denovo2413** |
| ***k__Bacteria; p__Firmicutes; c__Clostridia; o__Clostridiales; f__Veillonellaceae; g__Dialister*** |  |  |  |  |  |  |  |  | **1** |  | **1** | **46** | **420** | **321** | **115** | **denovo4692** |
| ***k__Bacteria; p__Firmicutes; c__Erysipelotrichi; o__Erysipelotrichales; f__Erysipelotrichaceae; g__RFN20*** | **22** | **2** | **2** |  |  |  |  |  | **359** | **264** |  |  |  |  |  | **denovo3932** |
| ***k__Bacteria; p__Proteobacteria; c__Alphaproteobacteria; o__Rhodospirillales; f__Acetobacteraceae; g__Acetobacter*** |  |  |  |  |  |  |  |  | **758** | **1502** |  |  |  |  | **1** | **denovo1302** |
| ***k__Bacteria; p__Proteobacteria; c__Gammaproteobacteria; o__Enterobacteriales; f__Enterobacteriaceae*** |  |  |  |  |  | **4** | **5** |  | **9** |  | **562** | **637** | **733** | **600** | **724** | **denovo4321** |

**Additional file 7: Figure S1.** Heat map: The abundances of 16S rRNA reads from the Dutch and Thai reactors (Filter by count per OTU: 500 per taxon) at genus-like level were presented. The date of sampling was shown (days). The numbers in each cell represented number of reads in each sample and OTU IDs were shown in the last column.
