# Supplementary material for: Organic acid production from potato starch waste fermentation by rumen microbial communities from Dutch and Thai dairy cows
Source: Biotechnol Biofuels. 2018 Jan 25;11:13. doi: 10.1186/s13068-018-1012-4 (PMC5784674; doi:10.1186/s13068-018-1012-4)
Supplement: Supplementary file 8 — Additional file 8: Table S7. The number of reads and OTUs per sample generated using 16S rRNA gene amplicon pyrosequencing from both reactors. [file 13068_2018_1012_MOESM8_ESM.docx]

***Figures, Tables and Additional files for Dutch and Thai manuscript***

**Organic acid production in potato starch waste fermentation by rumen microbial communities from Dutch and Thai dairy cows**

Susakul Palakawong Na Ayudthaya^1, 2^, Antonius H.P. van de Weijer^1^, Antonie H. van Gelder^1^, Alfons J. M. Stams^1,3^, Willem M. de Vos^1,4^ and Caroline M. Plugge^1*^

^1^Laboratory of Microbiology, Wageningen University & Research, Stippeneng 4, 6708 WE Wageningen, The Netherlands

^2^Thailand Institute of Scientific and Technological Research, 35 Mu 3, Khlong Ha, Amphoe Khlong Luang, Pathum Thani 12120 Thailand

^3^CEB-Centre of Biological Engineering, University of Minho, Campus de Gualtar, 4710-057 Braga, Portugal

^4^RPU Immunology, Department of Bacteriology and Immunology, University of Helsinki, Haartmaninkatu 3, FIN-00014 Helsinki, Finland

*Correspondence: [caroline.plugge@wur.nl](mailto:susakul.palakawongnaayudthaya@wur.nl),

Tel. + 31 (0) 317 483752

**Additional file 8: Table S7.** The number of reads and OTUs per sample generated using 16S rRNA gene amplicon pyrosequencing from both reactors

| **No. of reads (454-pyrosequencing)** | **Reactor** | | | | | | | | | | | | | |
| --- | --- | --- | --- | --- | --- | --- | --- | --- | --- | --- | --- | --- | --- | --- |
|  | **Dutch** | | | | | | | **Thai** | | | | | | |
|  | Dutch Inoculum | Sampling time (Days) | | | | | | Thai Inoculum | Sampling time (Days) | | | | | |
|  |  | 0.25 | 0.5 | 1.33 | 2 | 4 | 10 |  | 0 | 2 | 3 | 4 | 7 | 16 |
|  | 12,696 | 20,786 | 23,468 | 15,792 | 21,926 | 30,876 | 10,642 | 13,157 | 11,789 | 15,148 | 14,257 | 13,231 | 11,009 | 13,329 |
| **No. of OTUs** | 88 | 86 | 70 | 49 | 49 | 44 | 33 | 152 | 124 | 34 | 40 | 35 | 50 | 58 |
