# Supplementary material for: Organic acid production from potato starch waste fermentation by rumen microbial communities from Dutch and Thai dairy cows
Source: Biotechnol Biofuels. 2018 Jan 25;11:13. doi: 10.1186/s13068-018-1012-4 (PMC5784674; doi:10.1186/s13068-018-1012-4)
Supplement: Supplementary file 11 — Additional file 11: Figure S4. Principal component analysis (PCA) with unconstrained ordination triplot between the top 16 genus-like level phylogenetic groupings of the OTUs and the environmental variables explaining the variance with time in the Dutch (a) and Thai (b) reactors. Time points are indicated the sampling point (days) during the starch waste fermentation and shown as filled circles (●). Environmental variables or selected fermentation products are represented by red arrows. Bacterial community at genus-like groups with the level, i.e. phylum (P), order (O), family (F) or genus (G) are represented as blue arrows. The direction of the species, in which the species abundance increases. Length of arrows is a measure of fit. The environmental variable arrows (in red) approximated the correlation between species and an environmental variable. The further a product falls in the direction indicated by arrow, the higher the correlation. Both axes together explained 73 and 92.3% of the total variances in the datasets from the Dutch (a) and Thai (b) reactors, respectively. [file 13068_2018_1012_MOESM11_ESM.docx]

***Figures, Tables and Additional files for Dutch and Thai manuscript***

**Organic acid production in potato starch waste fermentation by rumen microbial communities from Dutch and Thai dairy cows**

Susakul Palakawong Na Ayudthaya^1, 2^, Antonius H.P. van de Weijer^1^, Antonie H. van Gelder^1^, Alfons J. M. Stams^1,3^, Willem M. de Vos^1,4^ and Caroline M. Plugge^1*^

^1^Laboratory of Microbiology, Wageningen University & Research, Stippeneng 4, 6708 WE Wageningen, The Netherlands

^2^Thailand Institute of Scientific and Technological Research, 35 Mu 3, Khlong Ha, Amphoe Khlong Luang, Pathum Thani 12120 Thailand

^3^CEB-Centre of Biological Engineering, University of Minho, Campus de Gualtar, 4710-057 Braga, Portugal

^4^RPU Immunology, Department of Bacteriology and Immunology, University of Helsinki, Haartmaninkatu 3, FIN-00014 Helsinki, Finland

*Correspondence: [caroline.plugge@wur.nl](mailto:susakul.palakawongnaayudthaya@wur.nl),

Tel. + 31 (0) 317 483752


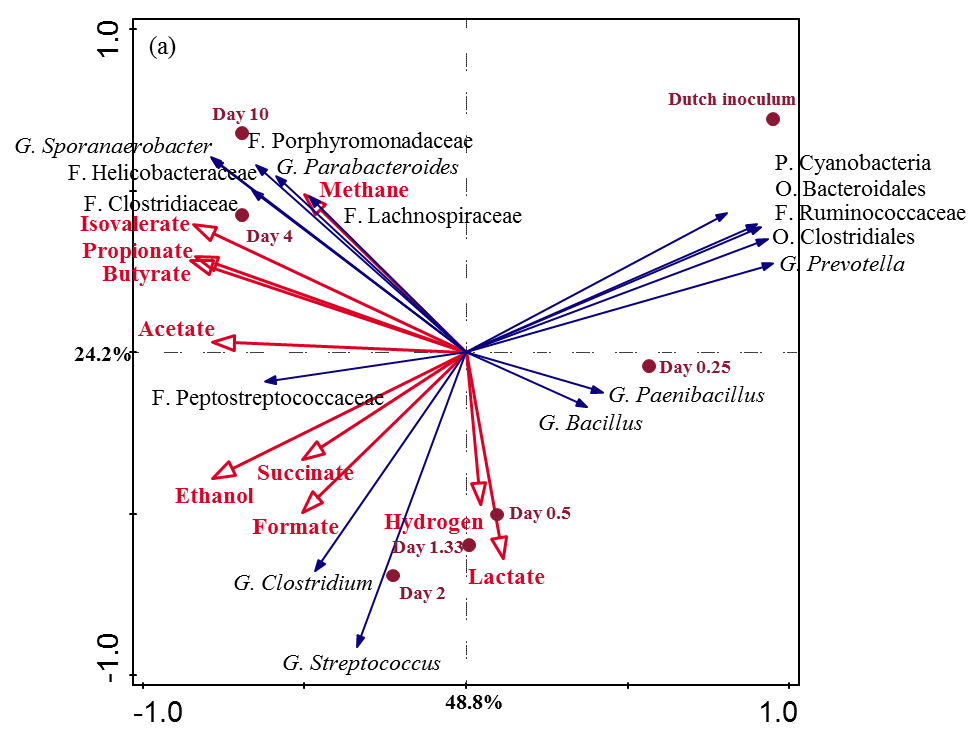


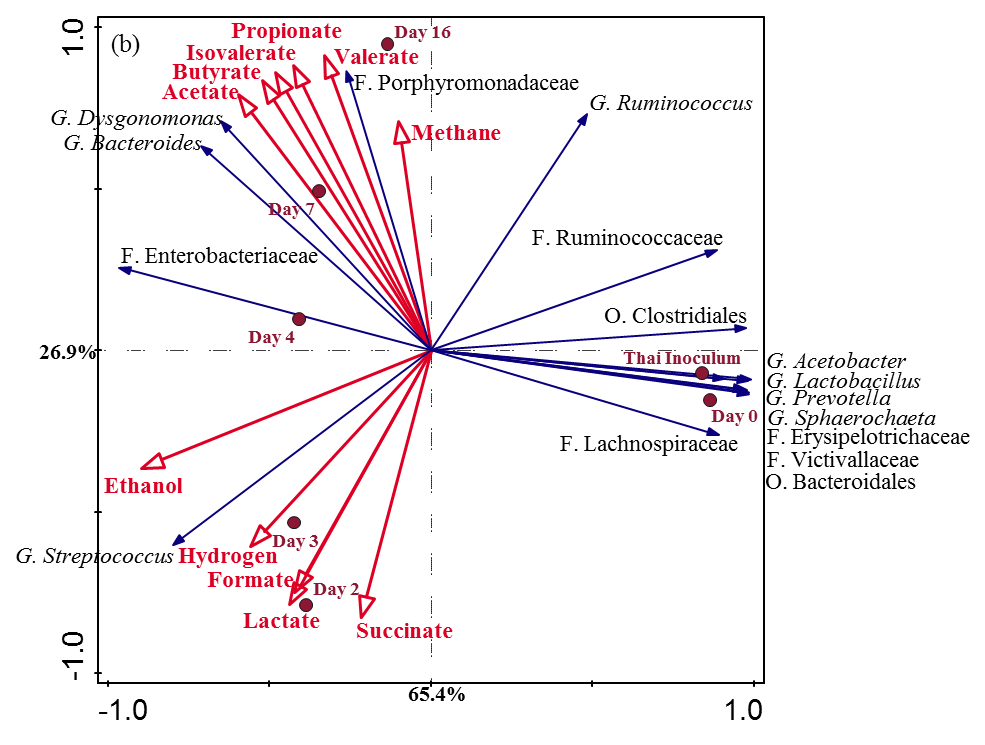


**Additional file 11: Figure S4**. Principal component analysis (PCA) with unconatrained ordination triplot between the top 16 genus-like level phylogenetic groupings of the OTUs and the environmantal variables explaining the variance with time in the Dutch (a) and Thai (b) reactors. Time points are indicated the sampling point (days) during the starch waste fermentation and shown as filled circles (●). Environmental variables or selected fermentation products are represented by red arrows. Bacterial community at genus-like groups with the level i.e. phylum (P), order (O), family (F) or genus (G) are represented as blue arrows. The direction of the species, in which the species abundance increases. Length of arrows is a measure of fit. The environmental variable arrows (in red) approximated the correlation between species and an environmental variable. The further a product falls in the direction indicated by arrow, the higher the correlation. Both axes together explained 73% and 92.3% of the total variances in the datasets from the Dutch (a) and Thai (b) reactors, respecitvely.
