# Supplementary material for: Organic acid production from potato starch waste fermentation by rumen microbial communities from Dutch and Thai dairy cows
Source: Biotechnol Biofuels. 2018 Jan 25;11:13. doi: 10.1186/s13068-018-1012-4 (PMC5784674; doi:10.1186/s13068-018-1012-4)
Supplement: Supplementary file 12 — Additional file 12: Table S8. Correlation matrix (Spearman’s Rank Order Correlations statistics) between Bacterial OTUs at genus-like level and the operational data from the Dutch reactor (a) and Thai reactor (b). Green colours indicate positive correlations, whereas red colours indicate negative correlations. Correlation is significant at the p = 0.05 level (2-tailed) for the groups in the solid parentheses, whereas the dashed parentheses indicate the significant correlations at p = 0.01 (2-tailed) and both font types are italic. [file 13068_2018_1012_MOESM12_ESM.docx]

***Figures, Tables and Additional files for Dutch and Thai manuscript***

**Organic acid production in potato starch waste fermentation by rumen microbial communities from Dutch and Thai dairy cows**

Susakul Palakawong Na Ayudthaya^1, 2^, Antonius H.P. van de Weijer^1^, Antonie H. van Gelder^1^, Alfons J. M. Stams^1,3^, Willem M. de Vos^1,4^ and Caroline M. Plugge^1*^

^1^Laboratory of Microbiology, Wageningen University & Research, Stippeneng 4, 6708 WE Wageningen, The Netherlands

^2^Thailand Institute of Scientific and Technological Research, 35 Mu 3, Khlong Ha, Amphoe Khlong Luang, Pathum Thani 12120 Thailand

^3^CEB-Centre of Biological Engineering, University of Minho, Campus de Gualtar, 4710-057 Braga, Portugal

^4^RPU Immunology, Department of Bacteriology and Immunology, University of Helsinki, Haartmaninkatu 3, FIN-00014 Helsinki, Finland

*Correspondence: [caroline.plugge@wur.nl](mailto:susakul.palakawongnaayudthaya@wur.nl),

Tel. + 31 (0) 317 483752

**Additional file 12: Table S8.** Correlation matrix (Spearman's Rank Order Correlations statistics) between Bacterial OTUs at genus-like level and the operational data from the Dutch reactor (a) and Thai reactor (b). Green colours indicate positive correlations, whereas red colours indicate negative correlations. Correlation is significant at the *p* = 0.05 level (2-tailed) for the groups in the solid parentheses, whereas the dashed parentheses indicate the significant correlations at *p* = 0.01 (2-tailed) and both font types are italic.

|  | Acetate | | Butyrate | | Lactate | | G. Bacillus | | G. Clostridium | | G. Paenibacillus | | | G. Parabacteroides | | G. Prevotella | | G. Sporanaerobacter | | G. Streptococcus | | F. Clostridiaceae | | F. Helicobacteraceae | | F. Lachnospiraceae | | F. Peptostreptococcaceae | | F. Porphyromonadaceae | | F. Ruminococcaceae | | O. Bacteroidales | | O. Clostridiales | | P. Cyanobacteria | |  |  |  |
| --- | --- | --- | --- | --- | --- | --- | --- | --- | --- | --- | --- | --- | --- | --- | --- | --- | --- | --- | --- | --- | --- | --- | --- | --- | --- | --- | --- | --- | --- | --- | --- | --- | --- | --- | --- | --- | --- | --- | --- | --- | --- | --- |
| Acetate |  | | *0.893* | | 0 | | -0.144 | | 0.5 | | -0.216 | | | *0.775* | | *-0.893* | | 0.591 | | 0.214 | | 0.25 | | 0.668 | | 0.286 | | 0.704 | | 0.591 | | *-0.821* | | *-0.821* | | *-0.821* | | *-0.865* | |  |  |  |
| Butyrate | *0.893* | |  | | -0.296 | | -0.324 | | 0.5 | | -0.324 | | | *0.883* | | *-1* | | *0.867* | | 0.107 | | 0.607 | | *0.802* | | 0.393 | | *0.852* | | *0.867* | | *-0.964* | | *-0.964* | | *-0.964* | | *-0.991* | |  |  |  |
| Lactate | 0 | | -0.296 | |  | | *0.86* | | 0.259 | | 0.748 | | | -0.58 | | 0.296 | | -0.654 | | *0.778* | | *-0.889* | | -0.647 | | -0.667 | | -0.308 | | -0.654 | | 0.296 | | 0.296 | | 0.296 | | 0.299 | |  |  |  |
| G. Bacillus | -0.144 | | -0.324 | | *0.86* | |  | | 0.054 | | *0.927* | | | -0.618 | | 0.324 | | -0.527 | | 0.595 | | -0.685 | | -0.584 | | -0.36 | | -0.337 | | -0.527 | | 0.27 | | 0.27 | | 0.27 | | 0.3 | |  |  |  |
| G. Clostridium | 0.5 | | 0.5 | | 0.259 | | 0.054 | |  | | -0.09 | | | 0.414 | | -0.5 | | 0.335 | | 0.643 | | 0.036 | | -0.045 | | -0.357 | | 0.667 | | 0.335 | | -0.571 | | -0.571 | | -0.571 | | -0.541 | |  |  |  |
| G. Paenibacillus | -0.216 | | -0.324 | | 0.748 | | *0.927* | | -0.09 | |  | | | -0.673 | | 0.324 | | -0.447 | | 0.595 | | -0.577 | | -0.449 | | -0.252 | | -0.486 | | -0.447 | | 0.234 | | 0.234 | | 0.234 | | 0.282 | |  |  |  |
| G. Parabacteroides | *0.775* | | *0.883* | | -0.58 | | -0.618 | | 0.414 | | -0.673 | | |  | | *-0.883* | | *0.875* | | -0.252 | | 0.739 | | *0.809* | | 0.541 | | *0.86* | | *0.875* | | *-0.847* | | *-0.847* | | *-0.847* | | *-0.873* | |  |  |  |
| G. Prevotella | *-0.893* | | *-1* | | 0.296 | | 0.324 | | -0.5 | | 0.324 | | | *-0.883* | |  | | *-0.867* | | -0.107 | | -0.607 | | *-0.802* | | -0.393 | | *-0.852* | | *-0.867* | | *0.964* | | *0.964* | | *0.964* | | *0.991* | |  |  |  |
| G. Sporanaerobacter | 0.591 | | *0.867* | | -0.654 | | -0.527 | | 0.335 | | -0.447 | | | *0.875* | | *-0.867* | |  | | -0.177 | | *0.906* | | *0.836* | | 0.611 | | *0.777* | | *1* | | *-0.906* | | *-0.906* | | *-0.906* | | *-0.895* | |  |  |  |
| G. Streptococcus | 0.214 | | 0.107 | | *0.778* | | 0.595 | | 0.643 | | 0.595 | | | -0.252 | | -0.107 | | -0.177 | |  | | -0.5 | | -0.356 | | -0.643 | | 0.037 | | -0.177 | | -0.179 | | -0.179 | | -0.179 | | -0.144 | |  |  |  |
| F. Clostridiaceae | 0.25 | | 0.607 | | *-0.889* | | -0.685 | | 0.036 | | -0.577 | | | 0.739 | | -0.607 | | *0.906* | | -0.5 | |  | | *0.757* | | 0.679 | | 0.593 | | *0.906* | | -0.643 | | -0.643 | | -0.643 | | -0.631 | |  |  |  |
| F. Helicobacteraceae | 0.668 | | *0.802* | | -0.647 | | -0.584 | | -0.045 | | -0.449 | | | *0.809* | | *-0.802* | | *0.836* | | -0.356 | | *0.757* | |  | | *0.757* | | 0.508 | | *0.836* | | *-0.757* | | *-0.757* | | *-0.757* | | *-0.787* | |  |  |  |
| F. Lachnospiraceae | 0.286 | | 0.393 | | -0.667 | | -0.36 | | -0.357 | | -0.252 | | | 0.541 | | -0.393 | | 0.611 | | -0.643 | | 0.679 | | *0.757* | |  | | 0.185 | | 0.611 | | -0.429 | | -0.429 | | -0.429 | | -0.414 | |  |  |  |
| F. Peptostreptococcaceae | 0.704 | | *0.852* | | -0.308 | | -0.337 | | 0.667 | | -0.486 | | | *0.86* | | *-0.852* | | *0.777* | | 0.037 | | 0.593 | | 0.508 | | 0.185 | |  | | *0.777* | | *-0.815* | | *-0.815* | | *-0.815* | | *-0.841* | |  |  |  |
| F. Porphyromonadaceae | 0.591 | | *0.867* | | -0.654 | | -0.527 | | 0.335 | | -0.447 | | | *0.875* | | *-0.867* | | *1* | | -0.177 | | *0.906* | | *0.836* | | 0.611 | | *0.777* | |  | | *-0.906* | | *-0.906* | | *-0.906* | | *-0.895* | |  |  |  |
| F. Ruminococcaceae | *-0.821* | | *-0.964* | | 0.296 | | 0.27 | | -0.571 | | 0.234 | | | *-0.847* | | *0.964* | | *-0.906* | | -0.179 | | -0.643 | | *-0.757* | | -0.429 | | *-0.815* | | *-0.906* | |  | | *1* | | *1* | | *0.991* | |  |  |  |
| O. Bacteroidales | *-0.821* | | *-0.964* | | 0.296 | | 0.27 | | -0.571 | | 0.234 | | | *-0.847* | | *0.964* | | *-0.906* | | -0.179 | | -0.643 | | *-0.757* | | -0.429 | | *-0.815* | | *-0.906* | | *1* | |  | | *1* | | *0.991* | |  |  |  |
| O. Clostridiales | *-0.821* | | *-0.964* | | 0.296 | | 0.27 | | -0.571 | | 0.234 | | | *-0.847* | | *0.964* | | *-0.906* | | -0.179 | | -0.643 | | *-0.757* | | -0.429 | | *-0.815* | | *-0.906* | | *1* | | *1* | |  | | *0.991* | |  |  |  |
| P. Cyanobacteria | *-0.865* | | *-0.991* | | 0.299 | | 0.3 | | -0.541 | | 0.282 | | | *-0.873* | | *0.991* | | *-0.895*  (a) | | -0.144 | | -0.631 | | *-0.787* | | -0.414 | | *-0.841* | | *-0.895* | | *0.991* | | *0.991* | | *0.991* | |  | |  |  |  |
|  | | | Acetate | | Butyrate | | Propionate | | Lactate | | G. Acetobacter | | G . Bacteroides | G. Dysgonomonas | | G. Pseudoramibacter_Eubacterium | | G. Lactobacillus | | G. Prevotella | | G. Sphaerochaeta | | G. Streptococcus | | F. Enterobacteriaceae | | F. Erysipelotrichaceae | | F. Lachnospiraceae | | F. Porphyromonadaceae | | F. Ruminococcaceae | | F. Victivallaceae | | O. Bacteroidales | | O. Clostridiales | | O. Lactobacillales |
| Acetate | | |  | | *1.000* | | *1.000* | | -0.374 | | -0.473 | | *0.964* | *0.929* | | *0.821* | | -0.286 | | *-0.906* | | *-0.757* | | 0.250 | | *0.857* | | *-0.802* | | *-0.929* | | *0.821* | | -0.286 | | -0.611 | | -0.414 | | -0.321 | | -0.250 |
| Butyrate | | | *1.000* | |  | | *1.000* | | -0.374 | | -0.473 | | *0.964* | *0.929* | | *0.821* | | -0.286 | | *-0.906* | | *-0.757* | | 0.250 | | *0.857* | | *-0.802* | | *-0.929* | | *0.821* | | -0.286 | | -0.611 | | -0.414 | | -0.321 | | -0.250 |
| Propionate | | | *1.000* | | *1.000* | |  | | -0.374 | | -0.473 | | *0.964* | *0.929* | | *0.821* | | -0.286 | | *-0.906* | | *-0.757* | | 0.250 | | *0.857* | | *-0.802* | | *-0.929* | | *0.821* | | -0.286 | | -0.611 | | -0.414 | | -0.321 | | -0.250 |
| Lactate | | | -0.374 | | -0.374 | | -0.374 | |  | | -0.196 | | -0.374 | -0.473 | | -0.473 | | -0.512 | | 0.185 | | -0.025 | | 0.512 | | -0.473 | | -0.147 | | 0.473 | | -0.571 | | -0.611 | | -0.304 | | -0.328 | | -0.611 | | -0.512 |
| G. Acetobacter | | | -0.473 | | -0.473 | | -0.473 | | -0.196 | |  | | -0.571 | -0.611 | | -0.709 | | *0.906* | | 0.685 | | *0.885* | | *-0.906* | | -0.611 | | *0.836* | | 0.512 | | -0.374 | | *0.867* | | 0.685 | | *0.875* | | *0.768* | | *0.906* |
| G. Bacteroides | | | *0.964* | | *0.964* | | *0.964* | | -0.374 | | -0.571 | |  | *0.964* | | *0.857* | | -0.357 | | *-0.906* | | *-0.757* | | 0.286 | | *0.821* | | *-0.802* | | *-0.857* | | *0.786* | | -0.321 | | -0.512 | | -0.450 | | -0.286 | | -0.286 |
| G. Dysgonomonas | | | *0.929* | | *0.929* | | *0.929* | | -0.473 | | -0.611 | | *0.964* |  | | *0.893* | | -0.393 | | *-0.867* | | *-0.802* | | 0.321 | | *0.857* | | *-0.757* | | *-0.893* | | *0.857* | | -0.286 | | -0.473 | | -0.414 | | -0.250 | | -0.321 |
| G. Pseudoramibacter_Eubacterium | | | *0.821* | | *0.821* | | *0.821* | | -0.473 | | -0.709 | | *0.857* | *0.893* | |  | | -0.393 | | *-0.867* | | *-0.802* | | 0.429 | | *0.964* | | *-0.757* | | *-0.893* | | 0.750 | | -0.393 | | -0.571 | | -0.631 | | -0.357 | | -0.429 |
| G. Lactobacillus | | | -0.286 | | -0.286 | | -0.286 | | -0.512 | | *0.906* | | -0.357 | -0.393 | | -0.393 | |  | | 0.473 | | *0.802* | | *-0.964* | | -0.321 | | *0.757* | | 0.286 | | -0.214 | | *0.929* | | 0.670 | | *0.757* | | *0.857* | | *0.964* |
| G. Prevotella | | | *-0.906* | | *-0.906* | | *-0.906* | | 0.185 | | 0.685 | | *-0.906* | *-0.867* | | *-0.867* | | 0.473 | |  | | *0.836* | | -0.473 | | *-0.867* | | *0.885* | | *0.867* | | -0.591 | | 0.512 | | 0.728 | | 0.716 | | 0.512 | | 0.473 |
| G. Sphaerochaeta | | | *-0.757* | | *-0.757* | | *-0.757* | | -0.025 | | *0.885* | | *-0.757* | *-0.802* | | *-0.802* | | *0.802* | | *0.836* | |  | | *-0.802* | | *-0.802* | | *0.944* | | *0.802* | | -0.668 | | *0.757* | | *0.836* | | *0.764* | | *0.757* | | *0.802* |
| G. Streptococcus | | | 0.250 | | 0.250 | | 0.250 | | 0.512 | | *-0.906* | | 0.286 | 0.321 | | 0.429 | | *-0.964* | | -0.473 | | *-0.802* | |  | | 0.393 | | *-0.757* | | -0.321 | | 0.179 | | *-0.964* | | *-0.768* | | *-0.847* | | *-0.929* | | *-1.000* |
| F. Enterobacteriaceae | | | *0.857* | | *0.857* | | *0.857* | | -0.473 | | -0.611 | | *0.821* | *0.857* | | *0.964* | | -0.321 | | *-0.867* | | *-0.802* | | 0.393 | |  | | *-0.757* | | *-0.964* | | *0.786* | | -0.357 | | -0.670 | | -0.595 | | -0.393 | | -0.393 |
| F. Erysipelotrichaceae | | | *-0.802* | | *-0.802* | | *-0.802* | | -0.147 | | *0.836* | | *-0.802* | *-0.757* | | *-0.757* | | *0.757* | | *0.885* | | *0.944* | | *-0.757* | | *-0.757* | |  | | *0.757* | | -0.579 | | *0.802* | | *0.885* | | *0.809* | | *0.802* | | *0.757* |
| F. Lachnospiraceae | | | *-0.929* | | *-0.929* | | *-0.929* | | 0.473 | | 0.512 | | *-0.857* | *-0.893* | | *-0.893* | | 0.286 | | *0.867* | | *0.802* | | -0.321 | | *-0.964* | | *0.757* | |  | | *-0.857* | | 0.286 | | 0.670 | | 0.468 | | 0.357 | | 0.321 |
| F. Porphyromonadaceae | | | *0.821* | | *0.821* | | *0.821* | | -0.571 | | -0.374 | | *0.786* | *0.857* | | 0.750 | | -0.214 | | -0.591 | | -0.668 | | 0.179 | | *0.786* | | -0.579 | | *-0.857* | |  | | -0.107 | | -0.394 | | -0.126 | | -0.143 | | -0.179 |
| F. Ruminococcaceae | | | -0.286 | | -0.286 | | -0.286 | | -0.611 | | *0.867* | | -0.321 | -0.286 | | -0.393 | | *0.929* | | 0.512 | | *0.757* | | *-0.964* | | -0.357 | | *0.802* | | 0.286 | | -0.107 | |  | | *0.808* | | *0.883* | | *0.964* | | *0.964* |
| F. Victivallaceae | | | -0.611 | | -0.611 | | -0.611 | | -0.304 | | 0.685 | | -0.512 | -0.473 | | -0.571 | | 0.670 | | 0.728 | | *0.836* | | *-0.768* | | -0.670 | | *0.885* | | 0.670 | | -0.394 | | *0.808* | |  | | *0.815* | | *0.906* | | *0.768* |
| O. Bacteroidales | | | -0.414 | | -0.414 | | -0.414 | | -0.328 | | *0.875* | | -0.450 | -0.414 | | -0.631 | | *0.757* | | 0.716 | | *0.764* | | *-0.847* | | -0.595 | | *0.809* | | 0.468 | | -0.126 | | *0.883* | | *0.815* | |  | | *0.847* | | *0.847* |
| O. Clostridiales | | | -0.321 | | -0.321 | | -0.321 | | -0.611 | | *0.768* | | -0.286 | -0.250 | | -0.357 | | *0.857* | | 0.512 | | *0.757* | | *-0.929* | | -0.393 | | *0.802* | | 0.357 | | -0.143 | | *0.964* | | *0.906* | | *0.847* | |  | | *0.929* |
| O. Lactobacillales | | | -0.250 | | -0.250 | | -0.250 | | -0.512 | | *0.906* | | -0.286 | -0.321 | | -0.429 | | *0.964* | | 0.473 | | *0.802* | | *-1.000* | | -0.393 | | *0.757* | | 0.321 | | -0.179 | | *0.964* | | *0.768* | | *0.847* | | *0.929* | |  |

(b)
